# Light‐Activated Ruthenium Nanoclusters Reprogram the Metabolic‐Quorum Sensing Axis for Precision Periodontitis Therapy

**DOI:** 10.1002/advs.76245

**Published:** 2026-06-22

**Authors:** Jing Zhou, Xiaolin Sun, Chengyu Liu, Yujia Shi, Meiqi Li, Meng Bo, Jingjie Zhai, Chunyan Li, Zhennan Wu, Xue Bai, Lin Wang

**Affiliations:** ^1^ Department of Oral Implantology Hospital of Stomatology Jilin Provincial Key Laboratory of Sciences and Technology for Stomatology Nanoengineering Jilin University Changchun China; ^2^ State Key Laboratory of Integrated Optoelectronics and College of Electronic Science and Engineering Jilin University Changchun China; ^3^ Department of Prosthodontics Jilin Provincial Key Laboratory of Tooth Development and Remodeling School and Hospital of Stomatology Jilin University Changchun China

**Keywords:** antibacterial therapy, bacterial metabolism, nanoclusters, periodontitis, photoelectrochemical catalysis, quorum sensing, ruthenium

## Abstract

Periodontitis is a polymicrobial disease driven by metabolic interdependencies, wherein commensal bacteria fuel the virulence of keystone pathogens like *Porphyromonas gingivalis* (*P. gingivalis*). In particular, *Veillonella parvula* (*V. parvula*) supplies essential nutrients to the keystone pathogen *P. gingivalis*, enabling its virulence even at low abundance. Therefore, targeting *V. parvula* nitrate metabolism to deprive *P. gingivalis* of nutrients and attenuate its virulence represents a promising therapeutic strategy for controlling periodontitis progression. We engineered L‐cysteine‐capped ruthenium nanoclusters (Ru NCs) that efficiently reduce nitrate to ammonium under 660 nm light. This photocatalytic conversion depleted the bioavailable nitrate pool associated with *V. parvula* metabolism, disrupting amino acid production and quorum‐sensing‐related support for *P. gingivalis*. Consequently, Ru NCs potently reduced biofilm biomass and thickness, suppressed *P. gingivalis* activity, and downregulated its key virulence genes *(RgpA/B*, *Kgp*, *FimA*). Multiomics analyses supported nitrate‐dependent metabolic collapse in *V. parvula*, which was associated with downstream energy and biosynthetic impairment in *P. gingivalis*. In a rat periodontitis model, light‐activated Ru NCs attenuated alveolar bone loss, preserved collagen, and shifted the local cytokine profile from pro‐inflammatory (IL‐6) to anti‐inflammatory (Arg‐1). This work provides a proof‐of‐concept strategy for targeting defined interspecies metabolic interactions, offering a new paradigm for microbiome‐targeted therapy.

## Graphical abstract

Under 660 nm visible light, Ru NCs efficiently photocatalyze nitrate‐to‐ammonium conversion and modulate oral nitrogen metabolism. They precisely perturb *Veillonella parvula*, block its key support to *Porphyromonas gingivalis*, and exert an “inhibiting without killing” antivirulence effect. Ultimately, this strategy corrects periodontitis‐driven dysbiosis, alleviates periodontal inflammation, and restores oral microbial homeostasis.

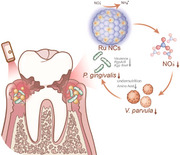

## 1 Introduction

Periodontitis is a dysbiotic, plaque‐associated inflammatory lesion that progressively destroys the tooth‐supporting tissues, ranking as the sixth most prevalent human disease and imposing formidable health‐economic burdens worldwide [1, 2, 3]. Currently, clinical management of periodontitis relies predominantly on mechanical debridement combined with adjunctive antibiotic therapy. However, this conventional paradigm is increasingly limited by its insufficient efficacy against bacteria embedded within complex biofilm architectures, high rates of disease recurrence, and the escalating threat of antimicrobial resistance [4]. Therefore, there is an urgent need to develop innovative therapeutic strategies that transcend traditional antimicrobial approaches.

In the pathogenesis of periodontitis, *Porphyromonas gingivalis* (*P. gingivalis*) is widely recognized as a keystone pathogenic species [1]. This organism produces a repertoire of virulence factors, including gingipains and lipopolysaccharide, which disrupt host immune homeostasis and drive dysbiotic inflammation [5]. Current therapeutic strategies targeting *P. gingivalis* primarily focus on reducing bacterial burden. However, a critical clinical paradox persists: even when treatment successfully suppresses *P. gingivalis* to extremely low levels, the residual population often retains a disproportionately strong capacity to promote disease recurrence [6, 7]. This paradox can be explained by the unique ecological strategy of *P. gingivalis* within the oral microbiome. *P. gingivalis*, even at sub‐threshold abundance, depends on the intercellular communication, regulating pathogen colonization and virulence expression [8]_._ Consequently, therapeutic strategies that target bacterial viability alone, without disrupting these keystone pathogenic interactions, are inherently insufficient. Growing evidence indicates that quorum sensing (QS), in concert with metabolic crosstalk, governs the stability, organization, and virulence output of periodontal pathogenic communities [9]. In this context, *Veillonella parvula* (*V. parvula*), an early colonizer and commensal species, has been shown to exert a significant influence on the growth and pathogenicity of *P. gingivalis* through its metabolic activities [10, 11]. During metabolism, *V. parvula* releases cell‐density dependent, growth‐promoting, soluble molecules that serve as nutritional substrates supporting *P. gingivalis* proliferation and virulence factor production [8]. Moreover, host‐derived inflammatory nitrate redirects *V. parvula* metabolism from lactate fermentation toward nitrate respiration, thereby doubling its biomass yield in lactate‐rich gingival crevicular fluid and expanding its biosynthetic capacity for peptide and heme precursors essential for *P. gingivalis* growth [12, 13]. Collectively, these findings suggest that disruption of this metabolic–QS axis represents a dual‐pronged therapeutic strategy, simultaneously depriving an obligate pathogen of critical nutrients and attenuating its virulence programming.

Nanomaterials provide a powerful platform for modulating microbial ecosystems by targeting multiple pathogenic processes, such as nutrient acquisition, metabolic crosstalk, and intercellular communication. In our previous work, we developed a triple‐responsive nanozyme platform (AgAu C‐L) that disrupts the “metabolic‐QS axis” between *F. nucleatum* and *P. gingivalis*, undermining biofilm integrity and pathogen support [14]. Similarly, Ni et al. designed acid‐responsive nanoparticles that block the electron transport chain while suppressing quorum sensing, coordinately inhibiting both metabolism and signaling [15]. Together, these studies highlight the potential of nanocatalytic medicine to intervene at the metabolic–QS axis that underlies periodontal microbial dysbiosis.

Despite these advances, the therapeutic efficacy of conventional nanomaterials against biofilm‐associated infections is frequently constrained by limited penetration through dense extracellular polymeric substances (EPS) and reduced bioavailability caused by particle aggregation [16, 17]. To overcome these challenges, ultra‐small nanoclusters (NCs) with diameters below 3 nm have emerged as a promising alternative. Their ultrasmall dimensions facilitate deep penetration into biofilms and rapid diffusion through EPS, while simultaneously providing a high catalytic surface area per unit mass [18]. Zheng et al. synthesized a series of uniformly ligand‐protected gold nanostructures spanning NCs to larger nanoparticles and demonstrated that only Au NCs were capable of penetrating bacterial cell walls, accumulating intracellularly, and inducing oxidative stress that disrupts essential metabolic processes [19]. Consistent with these findings, our group has shown that ultrasmall gold nanoclusters (AuNCs) can function as biofilm‐permeable, near‐infrared (NIR)‐activated photodynamic agents. Through atomic‐precision size control, AuNCs effectively infiltrated biofilms and internalized into bacterial cells, thereby suppressing biofilm formation and attenuating virulence‐associated pathways, including quorum sensing and transporter systems [20]. Among the diverse classes of metal NCs, ruthenium (Ru)‐based NCs are particularly attractive due to their exceptional catalytic activity in reducing nitrate (NO_3_^−^) to nitrite (NO_2_^−^) or ammonia (NH_3_) [21, 22]. This catalytic capability can be strategically exploited to disrupt the nitrate‐rich inflammatory microenvironment characteristic of periodontitis. By converting bioavailable nitrate into downstream reduced nitrogen species, Ru‐based nanocatalysts may weaken nitrate‐dependent metabolic support supplied by nitrate‐respiring species such as *V. parvula*, thereby attenuating *P. gingivalis* virulence within a defined pathogenic interaction.

**FIGURE 1:**
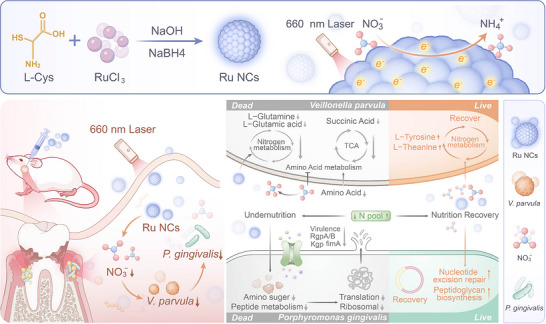
Schematic illustration of the design principle and biological applications of Ru NCs for regulating nitrate‐dependent metabolic crosstalk in a defined periodontal pathogenic model. Using L‐cysteine (L‐Cys) as a stabilizing ligand, Ru NCs were synthesized by reducing RuCl_3_ with NaBH_4_ under alkaline conditions. Upon excitation by 660 nm laser light, Ru NCs generated photoelectrons that promote the reduction of NO_3_^−^ to NH_4_^+^. In vitro and in vivo experiments demonstrated that this photocatalytic strategy inhibited *V. parvula*‐associated metabolic support and consequently suppressed the growth of *P. gingivalis* and its virulence factor expression (e.g., *RgpA/B, Kgp, fimA*), thereby demonstrating promising therapeutic efficacy against periodontitis. These effects are associated with nitrogen metabolic collapse and perturbation of amino acid metabolism in *V. parvula*, which was associated with metabolic depletion and translational repression in *P. gingivalis*.

In this study, we developed a Ru NCs–based photoelectrocatalytic strategy to disrupt nitrogen metabolic pathways in *V. parvula*, thereby reducing metabolic support for *P. gingivalis* and attenuating its virulence program (Figure 1). Ru NCs were synthesized via a solution‐phase reduction method using L‐cysteine (L‐Cys) as a stabilizing ligand. The resulting Ru NCs exhibited favorable physicochemical characteristics and robust electrochemical activity. Under visible‐light irradiation, Ru NCs directly inhibited the growth of *V. parvula* by perturbing its nitrogen metabolism and indirectly suppressed the proliferation of *P. gingivalis*. In parallel, Ru NCs treatment significantly downregulated key *P. gingivalis* virulence factors and adhesins. Moreover, Ru NCs remodeled the metabolic landscape of *V. parvula*, inducing amino acid depletion and nitrogen flux imbalance, which in turn drove transcriptional repression and metabolic collapse in downstream *P. gingivalis*. Finally, Ru NCs demonstrated pronounced therapeutic efficacy in an in vivo model of periodontitis. This strategy of disrupting the “metabolic‐QS axis” provides a proof‐of‐concept therapeutic pathway for periodontitis based on defined interspecies metabolic interference.

## 2 Results and Discussion

### 2.1 Synthesis and Characterization of Ru NCs

**FIGURE 2:**
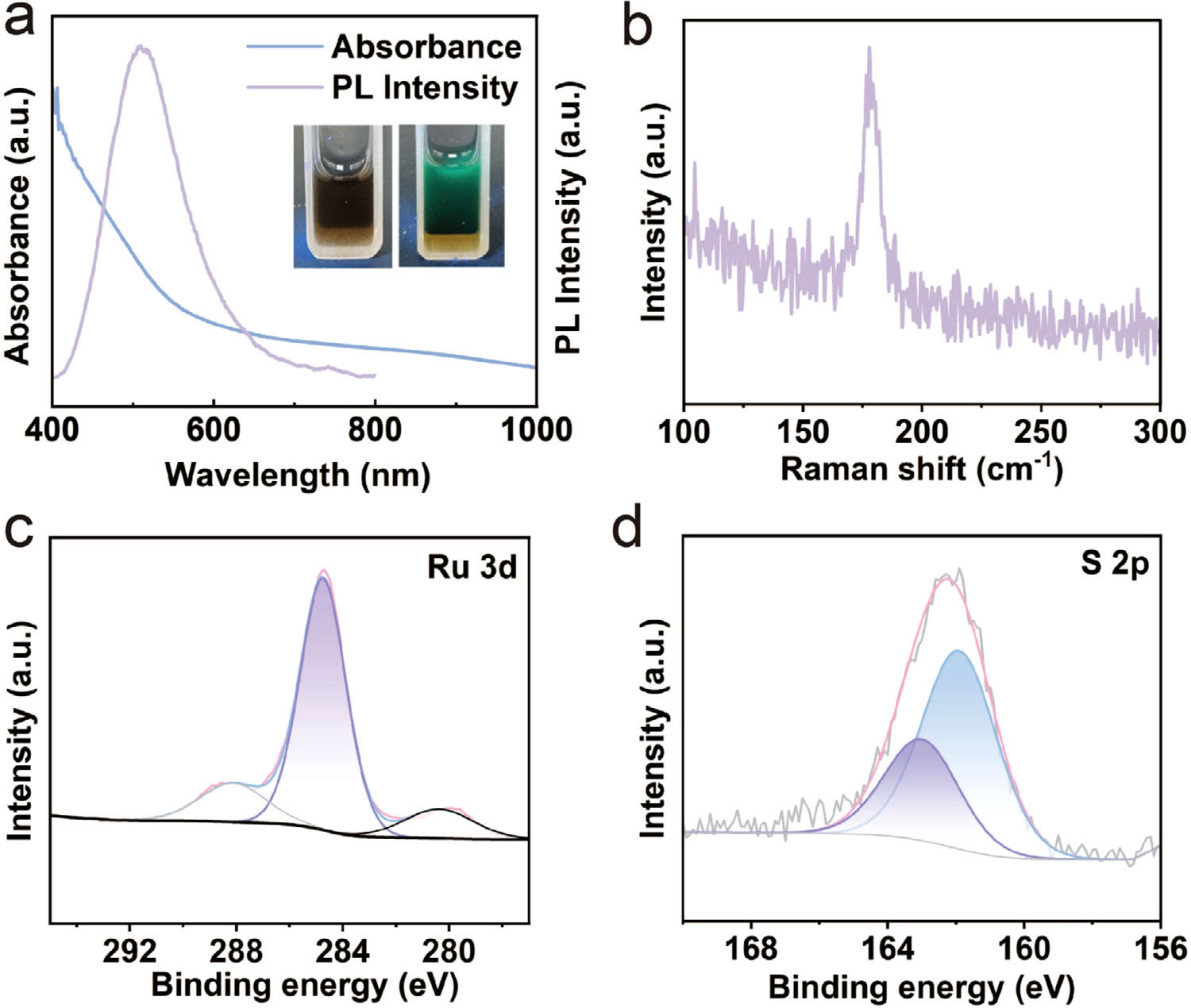
The preparation and characterization of Ru NCs. (a) UV–vis absorption spectra and photoluminescence (PL) spectrum of Ru NCs. (b) Raman spectroscopy of Ru NCs. (c‐d) XPS analysis of Ru NCs.

Ultrasmall Ru NCs were synthesized using L‐cysteine (L‐Cys) as a stabilizing ligand via a solution‐phase reduction route, following the schematic procedure illustrated in Figure S1a. L‐Cys was chosen because its thiol group stabilizes the ultrasmall clusters during synthesis, while its amino and carboxyl groups enhance hydrophilicity and biocompatibility, facilitating controlled nanocluster formation and potential biomedical applications. Molecular‐level purification was subsequently performed to obtain uniform nanoclusters. Transmission electron microscopy (TEM) images (Figure S1b) revealed well‐dispersed Ru NCs with an average particle diameter of approximately 2.05 nm, confirming the formation of ultrasmall and highly monodisperse nanoclusters. The UV–vis absorption spectrum of the Ru NCs (Figure 2a) exhibited a monotonically decreasing profile without distinct surface plasmon resonance peaks. A strong absorption band near 400 nm with gradual decay toward the near‐infrared region was observed, consistent with the characteristic featureless optical spectra reported for Ru NCs [23]. The photoluminescence (PL) spectrum (Figure 2a) displayed a strong and symmetric emission peak centered at approximately 500 nm, indicative of a uniform nanocluster size distribution and a single dominant emissive center [24, 25]. Fourier transform infrared (FTIR) spectroscopy (Figure S1c) was employed to investigate the coordination interaction between L‐Cys and the Ru NCs. The disappearance of the characteristic thiol (–SH) stretching vibration at approximately 2500 cm^−1^ indicated the formation of covalent Ru─S bonds [26, 27]. This coordination mode was further corroborated by Raman spectroscopy (Figure 2b), which revealed characteristic vibrational bands corresponding to Ru─Ru and Ru─S bonding [28]. X‐ray photoelectron spectroscopy (XPS) analysis (Figure 2c,d) provided additional insights into the chemical states of Ru and S. The Ru 3d and S 2p core‐level spectra exhibited binding‐energy shifts, with a prominent feature near ∼280 eV assigned to metallic Ru^0^, confirming the formation of Ru─Ru bonds and the partially reduced oxidation state of Ru within the nanoclusters [29].

### 2.2 Photothermal and Photoelectrocatalytic Properties of Ru NCs

**FIGURE 3:**
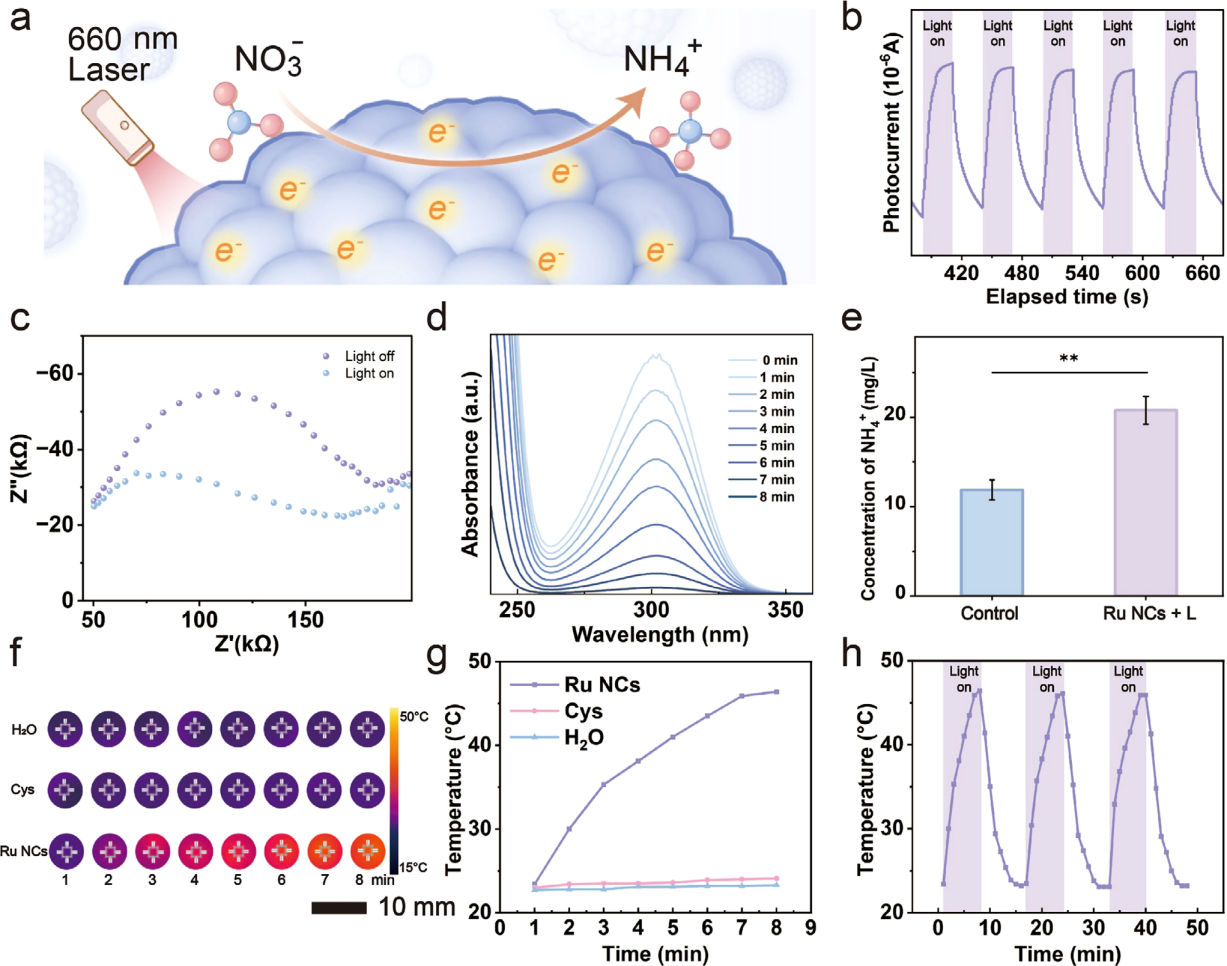
Photothermal and photoelectrocatalytic properties of Ru NCs. (a) Schematic representation of the photoelectrocatalytic process in Ru NCs. (b) Photocurrent response of Ru NCs under light irradiation. (c) Electrochemical impedance spectroscopy of Ru NCs. (d) UV–vis absorption spectra of nitrate solution under irradiation. (e) NH_4_^+^ concentration after nitrate reduction under light irradiation. (f) Photothermal imaging of H_2_O, Cys, and Ru NCs during laser irradiation. (g) Temperature versus time curve for Ru NCs. (h) Heating and cooling cycles under three irradiation periods. Data in quantitative panels are presented as mean ± SD (*n* = 3 independent experiments, unless otherwise indicated). Statistical significance was evaluated using one‐way ANOVA followed by Tukey's post‐hoc test; ^*^ *p* < 0.05, ^**^ *p* < 0.01, ^***^ *p* < 0.001; ns, not significant.

The catalytic mechanism is schematically illustrated in Figure 3a, wherein Ru NCs drive the photoreduction of nitrate (NO_3_^−^) to ammonium (NH_4_^+^) under 660 nm light irradiation. To elucidate the photoelectrocatalytic behavior, photoelectrochemical measurements were conducted to probe charge‐carrier dynamics. Upon periodic light on/off switching, Ru NCs exhibited pronounced and repeatable photocurrent responses (Figure 3b), indicating efficient photogenerated charge‐carrier generation and separation [30]. Complementary electrochemical impedance spectroscopy (EIS) analysis (Figure 3c) further revealed favorable charge‐transfer kinetics, supporting efficient interfacial charge transport within the Ru NCs system. Having established the photoelectrochemical activity, the photocatalytic reduction of NO_3_^−^ was subsequently investigated. UV–vis spectra collected over 0–8 min of light irradiation showed a progressive attenuation of the characteristic nitrate absorption band (Figure 3d), indicating rapid substrate consumption. Consistent with these observations, ion chromatography–mass spectrometry (IC–MS) quantification revealed a substantial increase in NH_4_^+^ concentration in the Ru NCs plus light (Ru NCs + L) group compared with control groups (Figure 3e), confirming the light‐driven conversion of nitrate to ammonium. Moreover, quantitative analysis of gingival crevicular fluid further confirmed that Ru NCs under 660 nm light effectively depleted local nitrate (from 619.57 to 277.31 mg/L) without causing ammonium‐associated cytotoxicity (Figure S2).

Notably, in addition to catalytic activity, Ru‐based nanomaterials have been reported to exhibit intrinsic photothermal conversion properties [31]. This photothermal effect acts as a “double‐edged sword” in therapeutic applications [32, 33]. On one hand, mild photothermal heating can synergistically enhance antibacterial efficacy and accelerate catalytic kinetics [32]. On the other hand, excessive hyperthermia exceeding biological tolerance limits risks causing irreversible thermal damage to surrounding healthy periodontal tissues [33]. Therefore, it is critical to evaluate whether the accompanying photothermal effect of Ru NCs remains within a safe and effective therapeutic window. To address this, the photothermal performance of Ru NCs was systematically assessed under 660 nm irradiation. As shown in the infrared thermal images (Figure 3f) and the corresponding temperature–time profiles (Figure 3g), Ru NCs rapidly increased the temperature of the dispersion to only about 50 °C (45.7 °C within 8 min), whereas L‐cysteine (Cys) and H_2_O exhibited negligible temperature changes under identical conditions. Notably, the photothermal response of Ru NCs remained highly reproducible over multiple light on/off cycles (Figure 3h), demonstrating excellent photothermal stability. This moderate temperature rise not only does not damage the material, but may even promote the catalytic reaction by enhancing molecular mobility and reaction kinetics.

To benchmark photostability under operational conditions, the absorbance profiles of Ru NCs and the reference photosensitizer chlorin e6 (Ce6) were monitored during continuous 660 nm irradiation (Figure S1d). Ru NCs maintained nearly constant absorbance over a 20 min period, whereas Ce6 exhibited a pronounced decline, demonstrating the superior photostability of Ru NCs under identical excitation conditions [34]. In addition, Ru NCs showed good structural and dispersion stability in artificial saliva over 7 days, as confirmed by UV–vis measurements (Figure S3), indicating that the material remains robust under simulated oral conditions. In contrast, nitrate solutions maintained in the dark over the same time interval exhibited negligible spectral changes (Figure S4), confirming that nitrate reduction does not proceed in the absence of light and validating the light‐dependent nature of the Ru NCs–mediated process. To exclude potential contributions from photodynamic effects, reactive oxygen species (ROS) generation was assessed using 1,3‐diphenylisobenzofuran (DPBF) as a probe under light irradiation (Figure S5). The DPBF absorbance remained unchanged over irradiation times of 0, 2, 4, and 9 min, indicating negligible ROS production by Ru NCs upon light exposure [35]. These findings suggest that the catalytic mechanism is dominated by photothermal and photoelectrochemical processes rather than ROS‐mediated pathways. Overall, the data support a stable, light‐driven catalytic system in which Ru NCs efficiently promote nitrate reduction without substantial ROS involvement.

Furthermore, evaluation of biocompatibility is a prerequisite for the biomedical application of Ru NCs. Consistent with this requirement, Ru NCs exhibited excellent biocompatibility and safety profiles. Treatment at different concentrations had limited effects on cell viability and morphology, with cells maintaining high survival rates and normal morphology within the 100–500 µg/mL range (Figure S6). To rule out potential thermal injury from the photothermal effect, hematoxylin and eosin (H&E) staining of the buccal mucosa adjacent to the treatment site under identical irradiation conditions revealed no tissue necrosis, inflammatory infiltration, or architectural disruption (Figure S7), confirming that local temperature elevation remained within a biosafe range. In vivo fluorescence imaging further demonstrated that Ru NCs were predominantly metabolized in the liver following tail vein injection, with no significant systemic retention observed within 24 h (Figure S8). No pathological alterations were observed in major organs (heart, liver, spleen, lung, kidney) at either 30 or 45 days post‐treatment (Figure S9), and key serum biochemical parameters remained within normal ranges (Figure S10). Collectively, these results demonstrate favorable clearance kinetics and short‐ and long‐term biosafety profiles, supporting the translational potential of this therapeutic strategy.

### 2.3 In Vitro Antibacterial Activity of Ru NCs

**FIGURE 4:**
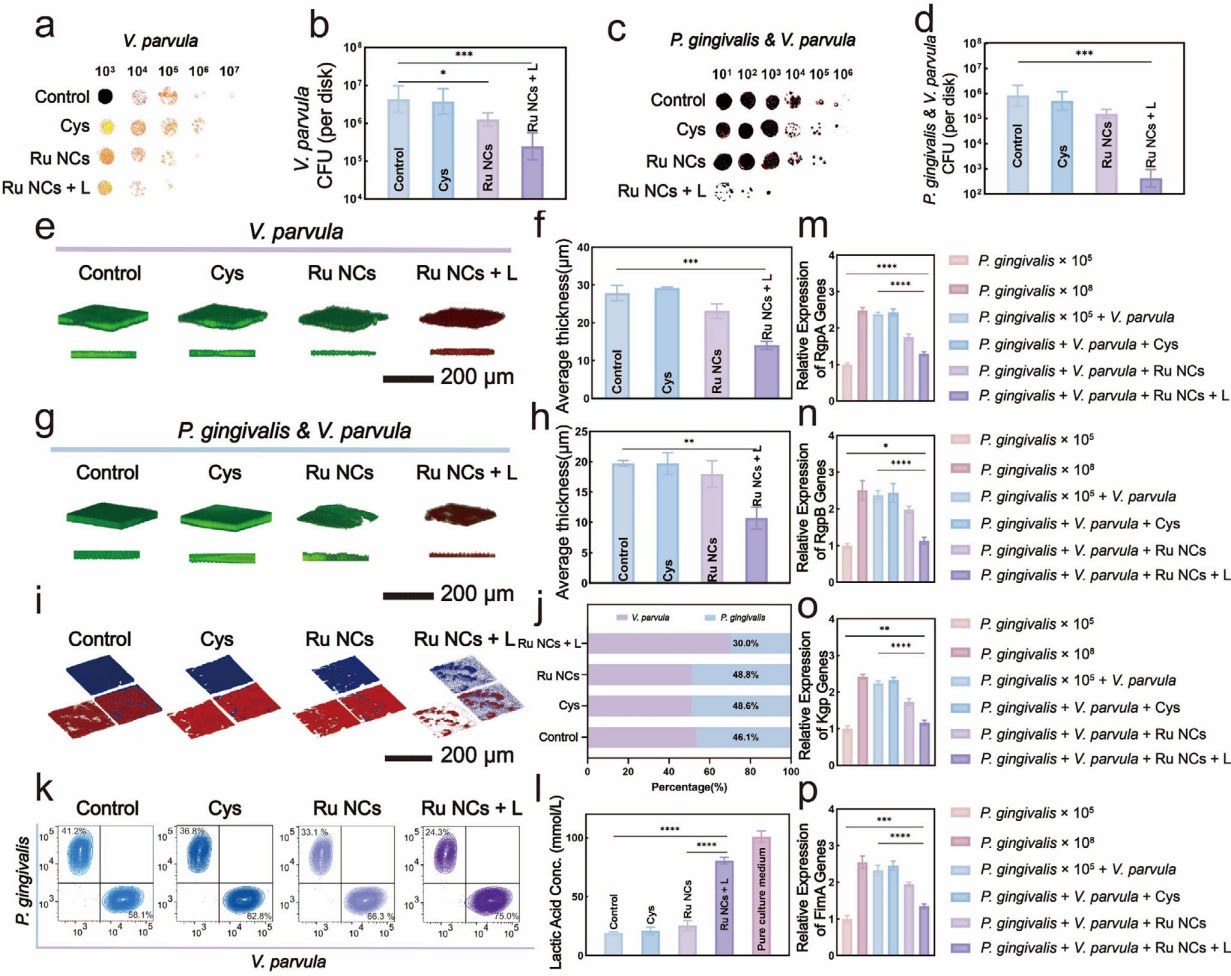
In vitro antibacterial performance of Ru NCs. (a) CFU images of *Veillonella parvula* (*V. parvula*) biofilm and (b) the corresponding statistical information. (c) CFU images of *Porphyromonas gingivalis* (*P. gingivalis*)&*V. parvula* biofilm and (d) the corresponding statistical information. (e) Representative three‐dimensional live/dead staining images and biofilm thickness in *V. parvula* biofilm. (f) Average thickness of *V. parvula* biofilms under different treatment conditions. (g) Representative three‐dimensional live/dead staining images and biofilm thickness in *V. parvula* and *P. gingivalis* biofilm. (h) Average thickness of *V. parvula* and *P. gingivalis* biofilms under different treatment conditions. (i) Biofilm FISH staining 3D image of *V. parvula* and *P. gingivalis* biofilm. (j) Statistical chart of the distribution of *V. parvula* and *P. gingivalis*. (k) Flow cytometry analysis of *V. parvula* and *P. gingivalis* biofilm. (l) Lactate levels after different treatments. (m‐p) PCR detection of *P. gingivalis* virulence genes (*RgpA/B, Kgp, fimA*). Data are presented as mean ± SD (*n* = 3 biological replicates or independent experiments). Statistical significance was evaluated using one‐way ANOVA followed by Tukey's post‐hoc test; ^*^ *p* < 0.05, ^**^ *p* < 0.01, ^***^ *p* < 0.001; ns, not significant. Scale bars are indicated in the corresponding images.

Single‐species *V. parvula* or *P. gingivalis* biofilms, as well as dual‐species *V. parvula–P. gingivalis* biofilms, were established to investigate the inhibitory effects of Ru NCs on *V. parvula* metabolism and the downstream consequences for *P. gingivalis*. As shown in Figure 4a–d and Figure S11, the Cys groups exhibited no significant reduction in colony‐forming units (CFUs) across either single‐species or dual‐species biofilms compared with untreated controls. Treatment with Ru NCs alone resulted in an approximately 1‐log reduction in *V. parvula* biofilms, whereas no statistically significant reduction was observed for *P. gingivalis* or the dual‐species biofilms, likely reflecting the intrinsically weak antibacterial activity of nanoclusters in the absence of external activation [36]. In contrast, upon 660 nm laser irradiation (Ru NCs + L group), the CFU count of *V. parvula* decreased by nearly two orders of magnitude, which can be attributed to the disruption of its nitrate metabolism mediated by the photoelectrocatalytic activity of Ru NCs. Under the same conditions, *P. gingivalis* exhibited an approximately 1‐log reduction, consistent with a predominantly photothermal inhibitory effect. Notably, the dual‐species *V. parvula–P. gingivalis* biofilms showed the most pronounced susceptibility, with CFU reductions of up to three orders of magnitude. Moreover, previous studies have shown that 660 nm light alone has no significant effect on the growth of periodontal pathogens; therefore, effective anti‐biofilm activity requires the combined activation of Ru NCs and light [37]. These findings indicate that light‐activated Ru NCs effectively disrupt the metabolic symbiosis between *V. parvula* and *P. gingivalis* by targeting nitrate‐dependent interactions, thereby amplifying antibacterial efficacy within the tested simplified biofilm model.

Live/dead confocal imaging revealed trends consistent with the CFU quantification results. Following Ru NCs treatment under light irradiation, single‐species *V. parvula* biofilms and dual‐species biofilms exhibited pronounced red fluorescence, indicative of extensive bacterial death. In contrast, single‐species *P. gingivalis* biofilms retained substantial green fluorescence, reflecting relatively higher bacterial viability. Quantitative biofilm thickness analysis further demonstrated that Ru NCs with irradiation markedly reduced biofilm architecture. Specifically, the thickness of dual‐species biofilms decreased from 19.71 µm in the control group to 10.72 µm after treatment, while single‐species *V. parvula* biofilms were reduced from 27.9 to 14.1 µm. In comparison, *P. gingivalis* biofilms showed a more modest reduction, from 36.93 µm in controls to 28.37 µm following treatment (Figure 4e–h and Figure S12).

To further elucidate interspecies interactions between *V. parvula* and *P. gingivalis*, fluorescence in situ hybridization (FISH) targeting both species, combined with flow cytometry analysis, was performed. In the control group, FISH imaging revealed a close spatial association between *P. gingivalis* and *V. parvula*, consistent with a symbiotic relationship characterized by tightly adjacent fluorescence signals. However, following Ru NC treatment with irradiation‐mediated nitrate degradation, the proportion of *P. gingivalis*–associated red fluorescence decreased from 46.1% in the control group to 30.0%, indicating disruption of this symbiotic association (Figure 4i,j). Flow cytometry analysis corroborated these findings, showing a reduction in *P. gingivalis* red fluorescence from 41.2% in the control group to 24.3% in the Ru NCs + L group (Figure 4k). In addition, to evaluate the selectivity of Ru NCs in a simplified mixed‐species setting, we performed FISH analysis on *Streptococcus gordonii (S. gordonii)*, a representative health‐associated oral commensal. In this model, Ru NCs + L treatment reduced the abundance of *V. parvula* (from 45% to 7%) and *P. gingivalis* (from 40% to 12%). In contrast, *S. gordonii* was largely unaffected and became the dominant species post‐treatment (81%) (Figure S13). These results indicate that Ru NCs + L preferentially reduced *V. parvula* and *P. gingivalis* while largely preserving *S. gordonii* under the tested conditions. However, this finding is based on a selected panel of species and does not represent the full complexity of the oral microbiome. A comprehensive microbiome‐wide assessment via 16S rRNA sequencing or metagenomic analysis will be pursued in future studies to further validate this selectivity across the broader oral microbiota.

Lactic acid, the primary carbon source utilized by *V. parvula*, was quantified to assess metabolic activity under different treatment conditions. The lactic acid concentration was approximately 101.05 mmol L^−1^ in pure culture medium and decreased to 19.15 mmol L^−1^ in the control group, reflecting active bacterial consumption. Following Ru NC treatment with irradiation, lactic acid levels increased significantly to 80.60 mmol L^−1^, indicating marked inhibition of *V. parvula* metabolic activity (Figure 4l).

To evaluate downstream effects on *P. gingivalis* virulence, the expression of key virulence‐associated genes (*RgpA*, *RgpB*, *Kgp*, and *FimA*) was quantified by PCR. As shown in Figure 4m–p, all four genes displayed consistent expression trends. Compared with the *P. gingivalis* × 10^5^ group, co‐culture with *V. parvula* (*P. gingivalis* × 10^5^ + *V. parvula*) resulted in significantly elevated virulence gene expression, approaching levels observed in the high‐density *P. gingivalis* × 10^8^ group, confirming the virulence‐amplifying effect of *V. parvula*. Similarly, virulence expression in the *P. gingivalis* + *V. parvula* + Cys group remained comparable to that of *P. gingivalis* × 10^8^. Although partial downregulation was observed in the *P. gingivalis* + *V. parvula* + Ru NCs group, expression levels remained higher than those of *P. gingivalis* × 10^5^ alone. Notably, virulence gene expression in the *P. gingivalis* + *V. parvula* + Ru NCs + L group was markedly reduced to levels comparable to the *P. gingivalis* × 10^5^ group. This pronounced suppression can be attributed to the disruption of *V. parvula* nitrate metabolism by light‐activated Ru NCs, thereby indirectly downregulating virulence programs in *P. gingivalis*.

### 2.4 In Vivo Antibacterial Properties of Ru NCs

**FIGURE 5:**
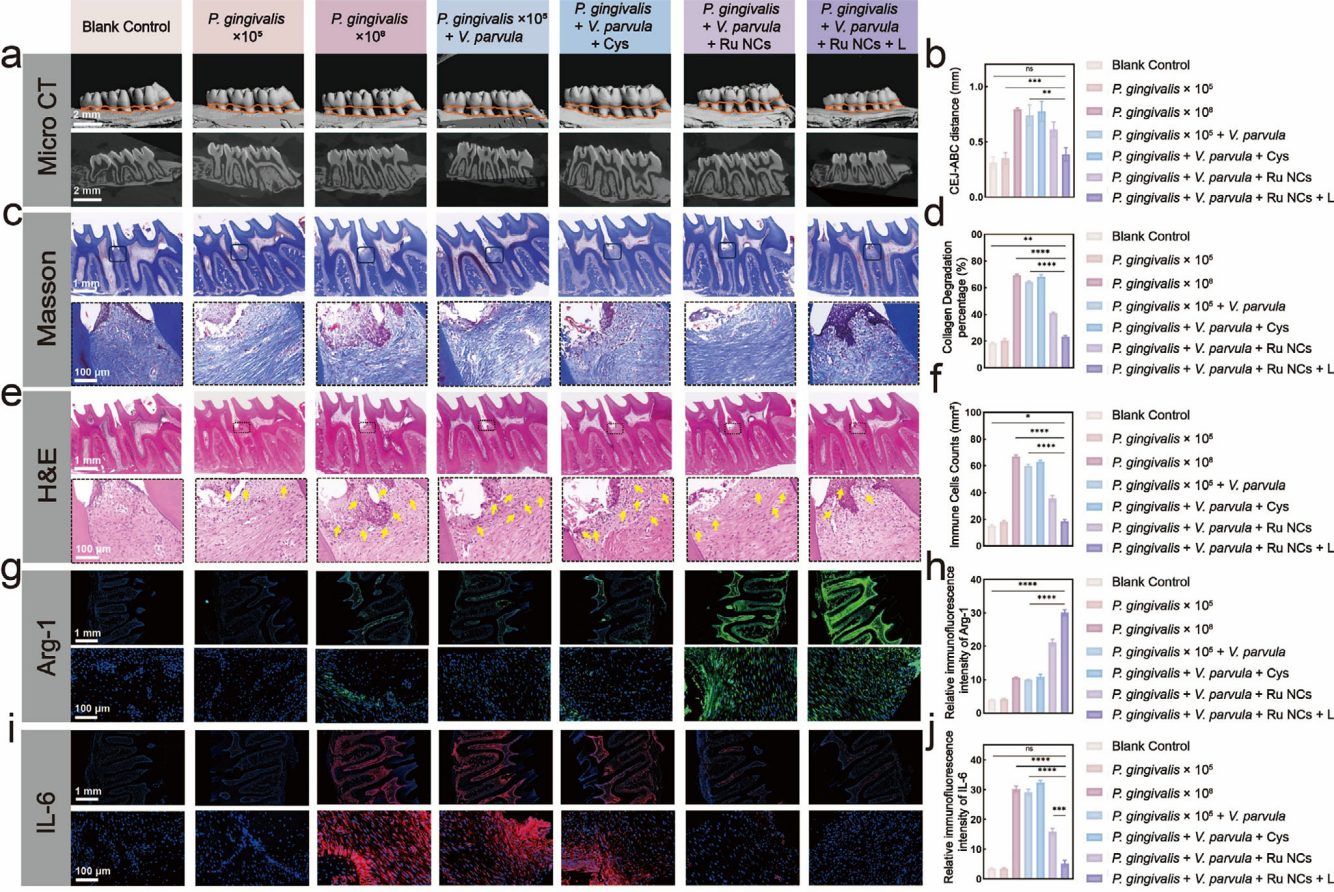
Treatment of periodontitis in vivo models using Ru NCs. (a,b) Three‐dimensional micro‐CT reconstruction and statistical analysis of maxillary molars. (c) Representative Masson's trichrome staining images. (d) Collagen degradation percentage in periodontal tissues. (e) Representative H&E staining images (yellow arrows: inflammatory cells). (f) Statistical analysis of immune cell numbers in periodontal tissues. (g,h) Arg‐1 Expression and Statistics. (i,j) IL‐6 Expression and Statistics. Data are presented as mean ± SD (*n* = 3 rats per group). Statistical significance was evaluated using one‐way ANOVA followed by Tukey's post‐hoc test; ^*^ *p* < 0.05, ^**^ *p* < 0.01, ^***^ *p* < 0.001; ns, not significant. Scale bars: 2 mm in micro‐CT images; 1 mm in low‐magnification histological and immunofluorescence images; 100 µm in high‐magnification histological and immunofluorescence images.

A rat model of periodontal infection was established to evaluate the in vivo anti‐infective efficacy of Ru NCs under 660 nm laser irradiation. After 14 days of therapeutic intervention, maxillary molars were subjected to three‐dimensional reconstruction using micro‐computed tomography (micro‐CT) (Figure 5a). Alveolar bone resorption was quantitatively assessed by measuring the distance from the cementoenamel junction (CEJ) to the alveolar bone crest (ABC) (Figure 5b). Compared with the blank control group (0.314 ± 0.015 mm), the *P. gingivalis* × 10^5^ group (0.351 ± 0.052 mm) exhibited no statistically significant difference, indicating that *P. gingivalis* at this inoculum did not induce marked alveolar bone loss. In contrast, both the *P. gingivalis* × 10^8^ group (0.793 ± 0.013 mm) and the *P. gingivalis ×* 10^5^ + *V. parvula* group (0.740 ± 0.091 mm) showed pronounced alveolar bone resorption. This result suggests that *V. parvula* markedly amplifies the pathogenic potential of low‐abundance *P. gingivalis*, enabling bone‐destructive effects comparable to those observed at high *P. gingivalis* inocula. Compared with the *P. gingivalis* × 10^8^ group, the *P. gingivalis* + *V. parvula* + Cys group showed no appreciable improvement in alveolar bone loss. Treatment with Ru NCs alone (*P. gingivalis* + *V. parvula* + Ru NCs group) resulted in a modest reduction in bone resorption, likely attributable to the limited intrinsic antibacterial activity of Ru NCs. Notably, the *P. gingivalis* + *V. parvula* + Ru NCs + L group exhibited the most pronounced protective effect, with a CEJ–ABC distance of 0.386 ± 0.061 mm, approaching that of the blank control. This substantial attenuation of bone loss can be attributed to the light‐activated electrocatalytic activity of Ru NCs, which disrupts nitrate metabolism in *V. parvula*, thereby indirectly suppressing *P. gingivalis* proliferation and virulence. Because an independent light‐only group was not included in the present in vivo study, the contribution of light irradiation alone cannot be fully excluded. However, previous reports indicated that 660 nm light alone has minimal antibacterial effects on periodontal pathogens under comparable conditions [37]. Therefore, the therapeutic benefit observed here should be interpreted primarily based on the direct comparison between the Ru NCs and Ru NCs + L groups. To further assess therapeutic efficacy at the tissue level, Masson's trichrome staining was performed. As shown in Figure 5c,d, the blank control group displayed densely distributed, well‐organized collagen fibers. In contrast, the *P. gingivalis* × 10^8^ group exhibited severe collagen degradation and fiber disorganization. Strikingly, collagen morphology and distribution in the *P. gingivalis* + *V. parvula* + Ru NCs + L group closely resembled those of the blank control, indicating effective preservation of periodontal connective tissue. These protective effects are likely mediated by Ru NCs–driven nitrate decomposition under laser irradiation, which perturbs *V. parvula* metabolism and consequently reduces *P. gingivalis*‐induced destruction of periodontal bone and soft tissues.

Moreover, H&E staining and immunofluorescence analysis were performed to further evaluate the therapeutic efficacy of Ru NCs under light irradiation. In the *P. gingivalis* × 10^8^ group, pronounced infiltration of inflammatory cells, including neutrophils and lymphocytes, was observed, indicating a robust host inflammatory response to bacterial challenge. In contrast, the *P. gingivalis* + *V. parvula* + Ru NCs + L group exhibited the lowest level of inflammatory cell infiltration within periodontal tissues, comparable to that of the blank control group, suggesting effective attenuation of inflammation (Figure 5e). Quantitative analysis (Figure 5f) further confirmed that the number of infiltrating inflammatory cells in the Ru NCs + L–treated group was significantly lower than in all other treatment groups, demonstrating a strong in vivo anti‐inflammatory effect. The anti‐inflammatory marker arginase‐1 (Arg‐1) was visualized by green fluorescence, whereas the pro‐inflammatory cytokine interleukin‐6 (IL‐6) was visualized by red fluorescence. In the *P. gingivalis* × 10^8^ group, intense red fluorescence was detected, reflecting elevated IL‐6 expression. In striking contrast, the *P. gingivalis* + *V. parvula* + Ru NCs + L group exhibited minimal red fluorescence accompanied by prominent green fluorescence, indicating upregulated Arg‐1 expression (Figure 5g,i). Quantitative analysis of these inflammatory markers revealed a significant increase in Arg‐1 expression and a concomitant decrease in IL‐6 expression in the Ru NCs + L–treated group (Figure 5h,j). Collectively, these results demonstrate that Ru NCs combined with light irradiation effectively reprogram the inflammatory response in periodontal tissues toward an anti‐inflammatory phenotype in vivo.

### 2.5 Ru NCs Reprogram *V. parvula* Amino Acid Metabolism and Nitrogen Utilization to Suppress *P. gingivalis* Transcription

**FIGURE 6:**
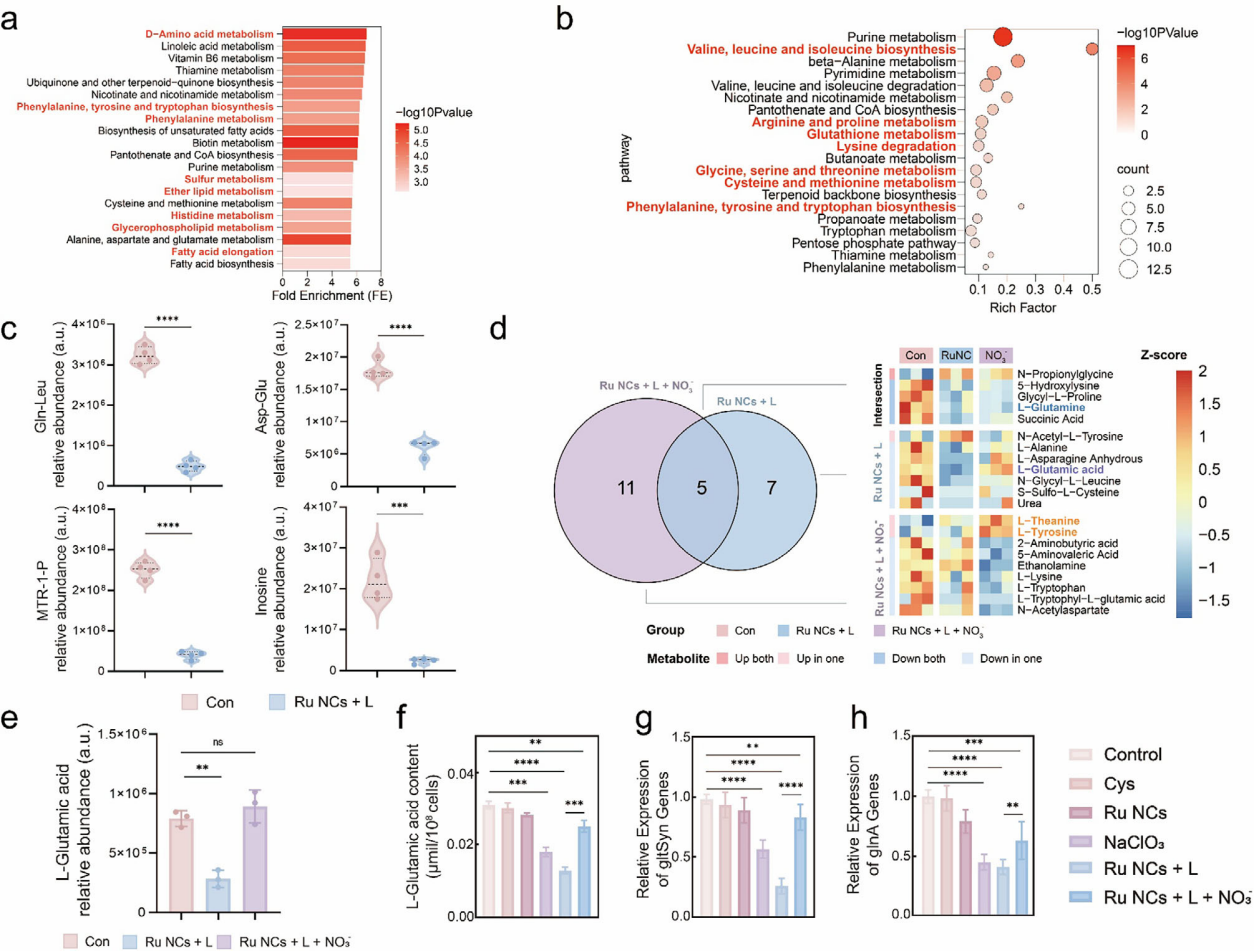
Non‐targeted and targeted metabolomic analysis of *V. parvula* under Ru NCs + L treatment with or without nitrate supplementation. Non‐targeted metabolomic analysis data (a) Quantitative enrichment analysis (QEA) of differential metabolites based on KEGG pathway annotation, highlighting significantly perturbed amino‐acid and nitrogen‐related pathways. (b) Over‐representation analysis (ORA) of downregulated metabolites identifies significantly enriched metabolic pathways associated with nitrogen utilization. (c) Violin plots showing the relative abundance of representative metabolites (Gln‐Leu, Asp‐Glu, MTR‐1‐P, and Inosine) in the Control and Ru NCs + L groups of *V. parvula*. Metabolite levels are presented as normalized LC‐MS peak areas in arbitrary units (a.u.). Data are shown as mean ± SD (n = 4 biological replicates); statistical significance was determined using Student's t‐test. (d) Venn diagram showing the overlap and unique differential metabolites among the three groups (Control group, Ru NCs + L group and Ru NCs +  L + NO_3_^−^ group), accompanied by a classification heatmap summarizing metabolite categories and regulation trends. (e) Relative abundance of key metabolites across the three groups, shown as normalized peak areas (a.u.). (f) Levels of L‐glutamic acid in *V. parvula* under different treatments. (g) Relative expression of *gltSyn* in *V. parvula* determined by RT‐qPCR. (h) Relative expression of *glnA* in *V. parvula* determined by RT‐qPCR. Data in panels (f–h) are presented as mean ± SD (*n* = 3 biological replicates); statistical significance was evaluated using one‐way ANOVA followed by Tukey's post‐hoc test; ^*^ *p* < 0.05, ^**^ *p* < 0.01, ^***^ *p* < 0.001; ns, not significant.

To elucidate the photoelectrocatalytic antibacterial mechanism of Ru NCs, the metabolomic and transcriptomic analyses were performed. Non‐targeted metabolomic analysis displayed the metabolic alterations of *V. parvula* following Ru NCs + L exposure. Principal component analysis (PCA) revealed a clear separation between the control and Ru NCs + L‐treated groups along PC1 (41.6%) and PC2 (26.8%), indicating significant metabolic remodeling induced by Ru NCs + L (Figure S14a). Volcano plot identified 395 differential metabolites, including 55 upregulated and 340 downregulated species (*p* < 0.05, VIP > 1, |log_2_FC| > 1; Figure S14b). Pathway enrichment analysis revealed that differential metabolites were mainly clustered in amino acid metabolism, including D‐amino acid metabolism; phenylalanine, tyrosine, and tryptophan biosynthesis; cysteine and methionine metabolism; histidine metabolism; and alanine, aspartate, and glutamate metabolism (Figure 6a). Perturbations were also observed in pantothenate and CoA biosynthesis, vitamin B6 metabolism, and thiamine metabolism, indicating disruption of cofactor and energy‐related processes linked to nitrogen utilization. KEGG analysis (Figure S14c) confirmed that amino acid metabolism was the most perturbed, accompanied by changes in purine metabolism and pantothenate and CoA biosynthesis, suggesting coupling between nitrogen metabolism and cellular energy production. Analysis of downregulated metabolites revealed marked suppression of valine, leucine, and isoleucine biosynthesis; arginine and proline metabolism; and glycine, serine, and threonine metabolism (Figure 6b). In contrast, only limited compensatory activation was observed in alanine, aspartate, and glutamate metabolism and glycine, serine, and threonine metabolism (Figure S14d), reflecting a “local compensation–global suppression” pattern typical of nutrient stress. Quantitative analysis of key nitrogen‐ and energy‐related metabolites showed significant reductions in Gln–Leu and Asp–Glu dipeptides, indicating impaired amino acid storage and transport [38, 39]. Decreased inosine levels suggested disrupted nucleotide and energy metabolism [40, 41], while lower 5′‐methylthioribose‐1‐phosphate (MTR‐1‐P) indicated inhibition of the methionine salvage pathway (Figure 6c) [42, 43]. Together, these results support a metabolic phenotype of “reduced amino acid supply–blocked nitrogen recycling–energy exhaustion” induced by Ru NCs + L.

To elucidate the impact of Ru NCs under light irradiation (Ru NCs + L) on amino acid metabolism and to clarify the regulatory contribution of nitrate, targeted amino acid metabolomics was further performed under three conditions: Control, Ru NCs + L, and Ru NCs + L + NO_3_^−^. PCA revealed clear metabolic separation among the three groups, with Ru NCs + L–treated samples distinctly segregated from controls, while nitrate supplementation shifted the metabolic profile toward an intermediate state (Figure S15a,b). Volcano plot analysis identified 12 differential metabolites between the Ru NCs + L and Control groups, of which 10 were downregulated (Figure S15c,e), and 16 differential metabolites between the Ru NCs + L + NO_3_^−^ and Control groups, including 13 downregulated metabolites (Figure S15d,f). Pathway enrichment analysis indicated that Ru NCs + L predominantly perturbed nitrogen metabolism, amino acid biosynthesis, energy metabolism, and redox homeostasis (Figure S15g). Heatmap analysis revealed marked reductions in L‐glutamine and urea levels, indicative of impaired nitrogen assimilation and recycling [44, 45]. Concurrent decreases in L‐alanine and L‐asparagine reflected suppression of amino acid biosynthetic capacity [46, 47], while reduced levels of glycyl‐L‐proline and N‐glycyl‐L‐leucine suggested disruption of peptide transport processes [48]. In addition, a decrease in succinic acid implied attenuation of tricarboxylic acid (TCA) cycle activity [49, 50], and reduced S‐sulfo‐L‐cysteine levels indicated compromised antioxidant defenses (Figure S15h) [51]. Collectively, these metabolic alterations define a characteristic “nitrogen limitation–energy depletion–oxidative imbalance” phenotype induced by Ru NCs + L in *V. parvula*. PCA and Venn analyses demonstrated that the Ru NCs + L + NO_3_^−^ group exhibited a metabolic pattern intermediate between the Control and Ru NCs + L groups (Figure S15i and Figure 6d). Several metabolites that were markedly depleted by Ru NCs + L treatment, including L‐glutamic acid, urea, L‐alanine, and L‐asparagine, were restored or modestly elevated upon nitrate addition. Metabolic network analysis identified four key metabolites—L‐glutamine, L‐glutamic acid, L‐tyrosine, and L‐theanine—as central responsive nodes (Figure S15j,k). L‐glutamine exhibited a pronounced decline following Ru NCs + L treatment with only partial recovery upon nitrate supplementation, whereas L‐glutamic acid showed a more robust rebound, suggesting partial reconstitution of nitrogen flux. The elevated levels of L‐tyrosine and L‐theanine reflected enhanced antioxidant capacity and adaptive metabolic responses (Figure 6e and Figure S15l–n). Together, these findings support a “nitrogen compensation–antioxidant enhancement–metabolic stabilization” model, in which nitrate supplementation mitigates Ru NCs + L‐induced metabolic stress but does not fully restore biosynthetic and energetic capacity in *V. parvula*. Of all the screened differential metabolites, L‐glutamic acid stood out as the core hub of nitrogen metabolism and displayed the most dramatic response to Ru NCs + L intervention. Therefore, we selected L‐glutamic acid as the representative marker to further validate the metabolic regulatory mechanism. As shown in Figure 6e, L‐glutamic acid content was markedly decreased under Ru NCs + L treatment and partially recovered after nitrate supplementation, confirming that the core nitrogen metabolism of *V. parvula* could be partially rescued by nitrate. To further verify the effect of Ru NCs + L on nitrogen metabolism, we re‐quantified the content of L‐glutamic acid using a colorimetric assay, and detected the expression levels of key metabolic genes *gltSyn* and *glnA via* RT‐qPCR. Notably, the nitrate reductase inhibitor NaClO_3_ alone could reduce L‐glutamic acid content and suppress the expression of related metabolic genes to a certain extent, yet its overall inhibitory efficacy was markedly weaker than Ru NCs + L treatment. By comparison, Ru NCs + L induced a much more significant decrease in L‐glutamic acid content and simultaneously downregulated the expression of the above key genes. In contrast, nitrate supplementation could partially rescue these phenotypic alterations caused by Ru NCs + L (Figure 6f–h). This indicates that the mechanism of action of Ru NCs + L is not limited to the inhibition of single enzyme activity, but induces the global reprogramming of the complex nitrogen metabolic network within *V. parvula* through photoelectrocatalysis. This multi‐target systemic intervention endows it with superior bioactivity in cutting off the nutrient supply of pathogenic bacteria and attenuating their virulence, compared with traditional chemical inhibitors. Notably, *V. parvula* is an obligate nitrate‐respiring bacterium that relies on NO_3_^−^ as both its essential terminal electron acceptor for anaerobic respiration and its primary nitrogen source. Critically, based on prior reports [12], *V. parvula* is thought to possess a limited capacity for NH_4_^+^ assimilation, which supports the interpretation that the photocatalytic conversion of NO_3_^−^ to NH_4_^+^ functionally depletes bioavailable nitrogen for this bacterium. Consistent with this notion, in vivo data showed that NH_4_^+^ levels in the periodontal pocket exhibited no significant accumulation despite active NO_3_^−^ reduction (Figure S2), which may be attributable to the alkaline microenvironment of inflamed pockets (pH > 7.5), where NH_4_^+^ deprotonates to volatile NH_3_ and dissipates. Consequently, Ru NCs + L induces nitrogen limitation through a dual mechanism: (i) deprivation of NO_3_^−^ as both electron acceptor and nitrogen source, and (ii) generation of NH_4_^+^ that may be less efficiently assimilated by *V. parvula* and may not be stably retained in the local niche under the tested inflammatory microenvironment. This species‐specific metabolic vulnerability underlies the efficacy of Ru NCs + L in disrupting *V. parvula* physiology.

**FIGURE 7:**
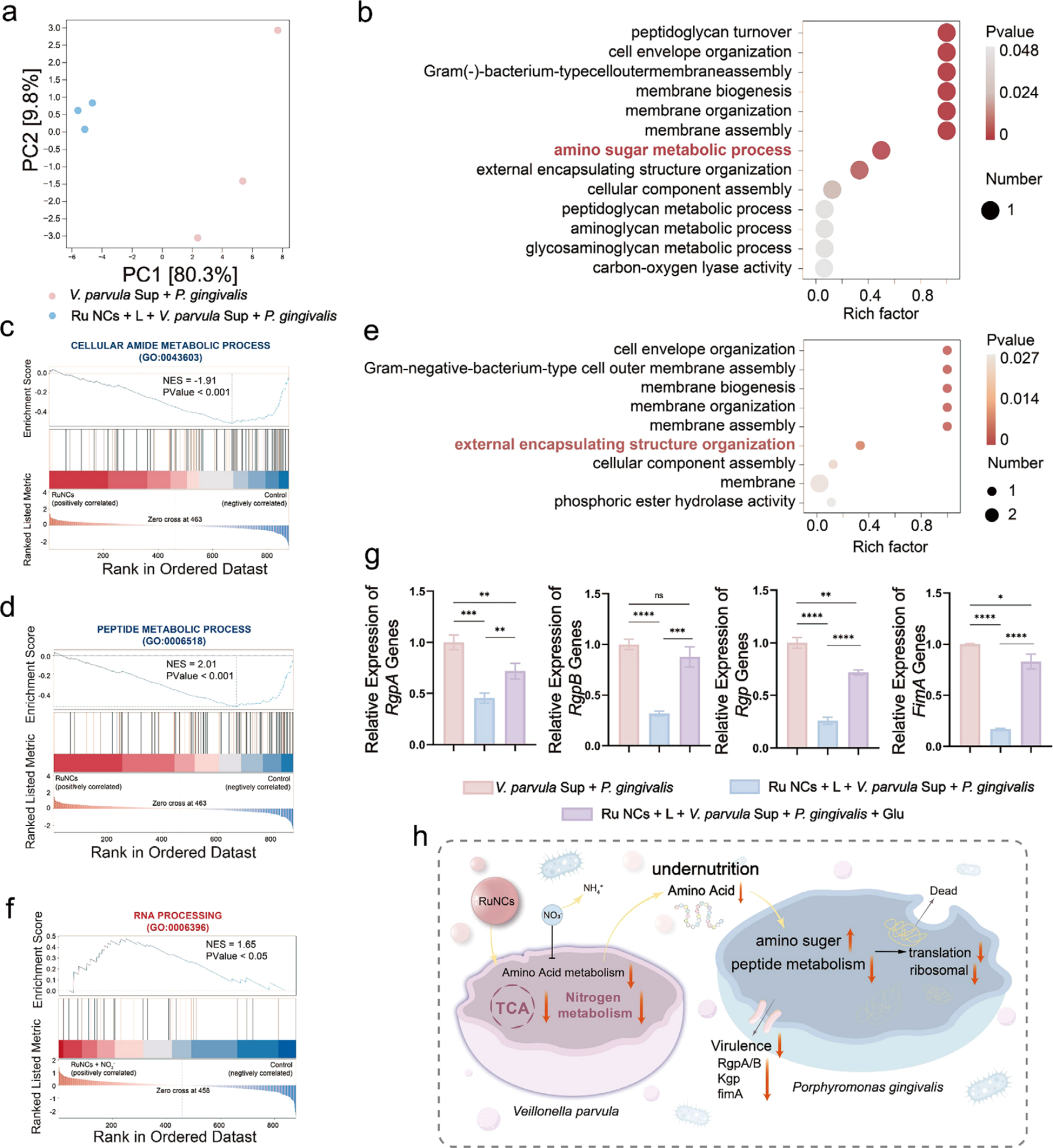
Transcriptomic analysis of *P. gingivalis* in supernatants derived from *V. parvula* under Ru NCs + L treatment with or without nitrate supplementation. (a) PCA plots showing transcriptional profiles of the Control and Ru NCs + L groups. (b) Gene Ontology (GO) enrichment analysis of upregulated gene sets in the Ru NCs + L‐treated group, highlighting envelope organization and translational repression. (c‐d) Gene Set Enrichment Analysis (GSEA) plots showing enrichment of representative biological processes in the Ru NCs + L‐treated group. (e) GO enrichment analysis of upregulated gene sets in the Ru NCs + L + NO_3_^−^ group, indicating enhanced membrane and structural assembly. (f) GSEA of representative pathways in the Ru NCs + L + NO_3_^−^ group illustrating partial recovery of transcriptional activity. (g) Rescue of *P. gingivalis* virulence gene expression by exogenous L‐glutamate. Relative expression levels of *P. gingivalis* virulence genes *RgpA*, *RgpB*, *Kgp*, and *FimA* under different treatments. (h) Schematic illustration summarizing the proposed mechanism by which Ru NCs modulate *P. gingivalis* physiology through nitrate‐mediated metabolic interference in *V. parvula*. Transcriptomic analyses were performed with n = 3 biological replicates. Data in panel (g) are presented as mean ± SD (n = 3 biological replicates); statistical significance was evaluated using one‐way ANOVA followed by Tukey's post‐hoc test; ^*^ *p* < 0.05, ^**^ *p* < 0.01, ^***^ *p* < 0.001; ns, not significant.

To determine whether upstream metabolic perturbations in *V. parvula* propagate to downstream species, *P. gingivalis* was cultured in supernatants derived from *V. parvula* subjected to Control, Ru NCs + L, or Ru NCs + L+ NO_3_^−^ treatments, followed by transcriptomic analysis. PCA revealed clear separation among treatment groups (Figure 7a and Figure S16a). Comparative analysis identified 67 differentially expressed genes (DEGs; 60 downregulated and 7 upregulated) between the Control and Ru NCs + L groups, and 37 DEGs (31 downregulated and 6 upregulated) between the Control and Ru NCs + L + NO_3_^−^ groups, with 29 DEGs shared between the two comparisons (Figure S16b–d). Gene Ontology (GO) enrichment analysis revealed that Ru NCs + L treatment upregulated genes associated with cell envelope organization, peptidoglycan turnover, and membrane biogenesis (Figure 7b), while strongly suppressing translation‐related functional categories, including ribosome, ribosomal subunit, structural constituent of ribosome, and peptide biosynthesis (Figure S16e). Consistent with these findings, gene set enrichment analysis (GSEA) demonstrated significant negative enrichment of cellular amide metabolic processes (NES = −1.91) and peptide metabolic processes (NES = −2.01) (Figure 7c,d). Protein–protein interaction (PPI) network analysis further revealed reduced connectivity among ribosomal proteins (e.g., RpmA, RpmB, and RpsT), indicating global translational repression (Figure S16f) [52, 53]. In addition, key oxidative stress–response genes, including *ahpC* and *ahpF*, were downregulated, suggesting compromised redox defense capacity [54]. Nitrate supplementation partially alleviated the transcriptional repression induced by Ru NCs + L. The total number of DEGs was reduced to 37 (Figure S16g), and the upregulated genes were primarily associated with cell envelope organization, membrane biogenesis, and structural assembly (Figure 7e). Notably, RNA processing pathways were positively enriched (NES = 1.65, *p* < 0.05; Figure 7f), while oxidative stress–related terms were diminished (Figure S16h), indicating partial relief of metabolic stress. Nevertheless, translation‐associated pathways remained significantly suppressed, suggesting that *P. gingivalis* persists in a low‐energy, low‐translation state despite nitrate supplementation. To further verify that the reduction of metabolites represented by L‐glutamic acid in *V. parvula* is the key driver of the attenuated virulence of downstream *P. gingivalis*, we performed an exogenous L‐glutamic acid rescue experiment using the nutrient‐deficient supernatant of *V. parvula* derived from the Ru NCs + L treatment group. The results showed that exogenous L‐glutamic acid supplementation partially restored the expression levels of key virulence genes of *P. gingivalis* (including *RgpA*, *RgpB*, *Kgp*, and *fimA*), which were significantly suppressed due to nutrient deprivation in the supernatant (Figure 7g). These results support the interpretation that Ru NCs + L indirectly attenuates *P. gingivalis* virulence, at least in part, by limiting the supply of key metabolites such as L‐glutamic acid from *V. parvula*, rather than acting solely through direct bactericidal effects.

Integration of metabolomic and transcriptomic datasets revealed a coordinated mechanism by which Ru NCs + L disrupt nitrogen and amino acid metabolism in the bridging bacterium *V. parvula*, leading to nitrogen limitation and translational repression in the downstream pathogen *P. gingivalis* (Figure 7h). Although nitrate supplementation partially mitigated these effects by replenishing nitrogen pools and improving redox balance, it failed to fully restore biosynthetic capacity and energy metabolism. Collectively, these findings indicate that Ru NCs + L indirectly modulate microbial community structure and virulence by reprogramming nitrogen flux within a keystone metabolic intermediary.

## 3 Conclusions

In this study, we developed a novel Ru NCs–based photoelectrocatalytic strategy to selectively disrupt nitrogen metabolism in *V. parvula*, thereby attenuating its symbiotic interaction with *P. gingivalis* and ultimately reducing the virulence of periodontal biofilms. Upon light irradiation, Ru NCs efficiently catalyzed the reduction of nitrate to ammonium, inducing nitrogen limitation in *V. parvula*, suppressing its metabolic activity, and consequently inhibiting the growth and virulence‐associated gene expression of *P. gingivalis*. Integrated metabolomic and transcriptomic analyses revealed that Ru NCs triggered a system‐level metabolic collapse in *V. parvula*, characterized by impaired amino acid biosynthesis, disrupted nitrogen recycling, and energy depletion. These upstream perturbations propagated to *P. gingivalis*, leading to transcriptional repression of ribosomal components and key virulence determinants. Notably, nitrate supplementation partially reversed these effects, confirming that the antimicrobial activity of Ru NCs is primarily mediated through nitrate‐dependent metabolic interference rather than direct bactericidal mechanisms. In vivo validation using a rat periodontitis model further demonstrated that Ru NCs combined with 660 nm laser irradiation markedly reduced alveolar bone resorption, preserved collagen architecture, and alleviated inflammatory cell infiltration. Although an independent light‐only group was not included in the present in vivo study, previous reports suggest that 660 nm light alone has limited direct antibacterial effects on periodontal pathogens under comparable conditions [37]. Therefore, within the experimental comparisons performed here, the therapeutic benefit of Ru NCs + L is mainly supported by its superior efficacy compared with Ru NCs alone. These therapeutic benefits were accompanied by a pronounced shift in the local immune milieu toward an anti‐inflammatory phenotype, as evidenced by increased Arg‐1 expression and reduced IL‐6 levels. Therefore, these findings establish a proof of concept for targeting the “microbiome–metabolism–pathogenesis” axis through selective metabolic modulation. By disrupting interspecies metabolic cooperation rather than indiscriminately eradicating bacteria, Ru NCs represent a precision antimicrobial strategy that minimizes ecological disturbance toward certain commensals (e.g., *S. gordonii*) and may reduce the risk of antimicrobial resistance. Nevertheless, the impact on the broader oral microbiome remains to be systematically investigated. This work thus provides a conceptual and technological framework for the development of next‐generation therapeutics against polymicrobial oral diseases such as periodontitis.

## 4 Experimental Section

### 4.1 Synthesis and Characterization of Ru NCs

Ru NCs were synthesized using L‐cysteine as the stabilizing ligand. Briefly, aqueous L‐cysteine solution was mixed with RuCl_3_ solution at a Ru/Cys molar ratio of 1:2, followed by pH adjustment to 10.5. Freshly prepared NaBH_4_ solution was added dropwise under ice‐bath conditions, and the reaction was allowed to proceed for more than 24 h. The resulting Ru NCs were purified by centrifugation, ethanol washing, freeze‐drying, and redispersion in deionized water. UV–vis absorption spectroscopy, photoluminescence spectroscopy, transmission electron microscopy, FTIR spectroscopy, Raman spectroscopy, and X‐ray photoelectron spectroscopy were used to characterize the optical properties, morphology, ligand coordination, and chemical states of Ru NCs.

### 4.2 Photoelectrochemical and Photothermal Measurements

Photoelectrochemical measurements were performed using a three‐electrode system with a Ru NCs‐modified glassy carbon electrode as the working electrode, a platinum wire as the counter electrode, and a saturated calomel electrode as the reference electrode. Photocurrent responses and electrochemical impedance spectroscopy were recorded under 660 nm light irradiation. Photocatalytic nitrate reduction was evaluated by monitoring nitrate consumption and NH_4_^+^ production. Photothermal performance was assessed by irradiating Ru NCs dispersions with a 660 nm laser and recording real‐time temperature changes using an infrared thermal imaging system.

### 4.3 In Vitro Biofilm Assays

*P. gingivalis* and *V. parvula* were cultured anaerobically in their corresponding culture media. Single‐species and dual‐species biofilms were established to evaluate the antibacterial effects of Ru NCs with or without 660 nm light irradiation. Biofilm viability and structure were analyzed by colony‐forming unit counting, live/dead staining, confocal laser scanning microscopy, fluorescence in situ hybridization, and flow cytometry. Lactate levels were measured to assess *V. parvula* metabolic activity. The expression of *P. gingivalis* virulence‐associated genes, including *RgpA*, *RgpB*, *Kgp*, and *fimA*, was quantified by RT‐qPCR.

### 4.4 Metabolomic and Transcriptomic Analyses

Untargeted metabolomics and targeted amino acid metabolomics were performed to evaluate metabolic alterations in *V. parvula* following Ru NCs + L treatment, with or without NaNO_3_ rescue. Differential metabolites were identified according to the criteria described in the Supporting Information, followed by KEGG pathway enrichment analysis. To assess downstream transcriptional effects, *P. gingivalis* was cultured with cell‐free supernatants from *V. parvula* subjected to different treatments, followed by RNA sequencing. Differentially expressed genes were analyzed using DESeq2, and functional enrichment was performed using GO and KEGG analyses.

### 4.5 Animal Experiments and Ethical Approval

All animal experiments were approved by the Institutional Animal Care and Use Committee of the College of Basic Medical Sciences, Jilin University, China (Approval No. 2025–692), and were conducted in accordance with relevant institutional and national guidelines for animal care and use. A rat periodontitis model was established by placing ligature wire around the cervical region of the maxillary second molar, followed by local bacterial inoculation. After model establishment, animals were randomly assigned to the indicated treatment groups, including blank control, *P. gingivalis* × 10^5^ group (10^5^ CFU/mL *P. gingivalis* without treatment), *P. gingivalis* × 10^8^ group (10^8^ CFU/mL *P. gingivalis* without treatment), *P. gingivalis* × 10^5^ + *V. parvula* group (10^5^ CFU/mL *P. gingivalis* and 10^8^ CFU/mL *V. parvula* without treatment), *P. gingivalis* + *V. parvula* + Cys group (10^5^ CFU/mL *P. gingivalis* and 10^8^ CFU/mL *V. parvula* treated with L‐cysteine), *P. gingivalis* + *V. parvula* + Ru NCs group (10^5^ CFU/mL *P. gingivalis* and 10^8^ CFU/mL *V. parvula* treated with Ru NCs), and *P. gingivalis* + *V. parvula* + Ru NCs + L group (10^5^ CFU/mL *P. gingivalis* and 10^8^ CFU/mL *V. parvula* treated with Ru NCs followed by 660 nm irradiation for 5 min). After 14 days of treatment, the maxillary tissues were harvested and fixed in 4% paraformaldehyde. Therapeutic efficacy was evaluated by micro‐computed tomography, H&E staining, Masson's trichrome staining, and immunofluorescence staining of inflammatory markers.

### 4.6 Statistical Analysis

Data are presented as mean ± standard deviation unless otherwise indicated. The number of biological replicates or independent experiments is specified in the corresponding figure legends. Statistical analyses were performed using SPSS 19.0 and Origin 2023. Comparisons between two groups were performed using Student's t‐test, whereas comparisons among multiple groups were analyzed by one‐way analysis of variance followed by Tukey's post‐hoc test. Statistical significance was defined as *p* < 0.05. The statistical thresholds are indicated as ^*^ *p* < 0.05, ^**^ *p* < 0.01, ^***^ *p* < 0.001, and ns, not significant.

## Author Contributions

**Jing Zhou**: Writing – review & editing, Writing – original draft, data curation. **Xiaolin Sun**: Writing – review & editing, Supervision. **Chengyu Liu**: Software, Methodology, Formal analysis, Data curation. **Yujia Shi**: Visualization, Software, Investigation. **Meiqi Li**: Writing – review & editing, Software, Methodology. **Meng Bo**: Software, Data curation. **Jingjie Zhai**: Data curation. **Chunyan Li**: Writing – review & editing, Supervision. **Zhennan Wu**: Conceptualization, Supervision. **Xue Bai**: Conceptualization, Supervision, Resources. **Lin Wang**: Writing – review & editing, Conceptualization, Supervision, Resources, Funding acquisition.

## Funding

This work was supported by the National Natural Science Foundation of China (82571148), Natural Science Foundation of Jilin Province (YDZJ202401209ZYTS), Science and Technology Project of Jilin Province Financial Department (JCSZ2025678‐4, ‐11, ‐15, ‐19), Interdisciplinary Innovation Team Project of Norman Bethune Health Science Department Jilin University (2025JBGS04).

## Conflicts of Interest

The authors declare no conflicts of interest.

## Supporting information




**Supporting File**: advs76245‐sup‐0001‐SuppMat.docx.

## Data Availability

The data that support the findings of this study are available from the corresponding author upon reasonable request.
