# Supplementary material for: Light‐Activated Ruthenium Nanoclusters Reprogram the Metabolic‐Quorum Sensing Axis for Precision Periodontitis Therapy
Source: Adv Sci (Weinh). 2026 Jun 22:e76245. Online ahead of print. doi: 10.1002/advs.76245 (PMC13336796; doi:10.1002/advs.76245)
Supplement: Supplementary file 1 — Supporting File: advs76245‐sup‐0001‐SuppMat.docx. [file ADVS-9999-e76245-s001.docx]

Supporting Information

**Light-Activated Ruthenium Nanoclusters Reprogram the Metabolic-Quorum Sensing Axis for Precision Periodontitis Therapy**

*Jing Zhou ^a^*, *Xiaolin Sun ^a^, Chengyu Liu ^c^, Yujia Shi ^a^, Meiqi Li ^a^, Meng Bo ^a^, Jingjie Zhai ^a^, Chunyan Li ^c^*, Zhennan Wu ^b^*, Xue Bai ^b^*, and Lin Wang ^a^**

**Contents of the Supplementary Information**

- Materials and Methods
- Additional **Figure S1-S16** and notes

**Materials and Methods**

**1. Materials**

Ruthenium(III) chloride (RuCl_3_), L-cysteine (L-Cys), sodium hydroxide (NaOH, 99.99%), and sodium borohydride (NaBH_4_) were purchased from Aladdin Reagent Co., Ltd. (Shanghai, China). Ultrapure Millipore water (18.2 MΩ·cm) was used to dissolve reagents and as the reaction medium. All chemicals were used as received without further purification.

Reagents for cell culture included penicillin–streptomycin solution (Hyclone, Logan, UT, USA). Dulbecco’s Modified Eagle Medium (DMEM) and fetal bovine serum (FBS) were obtained from Gibco (Grand Island, NY, USA). Tryptic Soy Broth (TSB), yeast extract, L-cysteine hydrochloride, hemin, and vitamin K were supplied by Sigma-Aldrich (USA). Mouse fibroblast cell line L929 was obtained from the American Type Culture Collection (ATCC, USA). Dulbecco’s phosphate-buffered saline (PBS) and other analytical reagents (Adamas-beta®) were purchased from Titan Scientific Co., Ltd. (China). Culture media and supplements for *Veillonella* species were obtained from Shandong Topbio Co., Ltd.

1. **Methods**

**2.1 Synthesis of Ru NCs**

Details of the synthesis of Ru NCs are provided in the main text.

**2.2 Characterization of Ru NCs**

After the successful synthesis of Ru NCs, ultraviolet–visible (UV–Vis) absorption spectra were recorded using a Shimadzu UV-1900i spectrophotometer. Photoluminescence (PL) spectra were obtained on a Hitachi F-4700 fluorescence spectrometer with both excitation and emission slit widths set to 2.5 nm. A xenon lamp with a fixed excitation wavelength of 405 nm was employed as the excitation source. Transmission electron microscopy (TEM) images were acquired using a FEI Tecnai G2 F20 microscope operated at an accelerating voltage of 200 kV.

The vibrational modes of L-cysteine (L-Cys) and ruthenium nanoclusters (Ru NCs) were characterized by Fourier transform infrared (FTIR) spectroscopy (Billerica, MA, USA). Raman spectra were collected using a high-resolution laser Raman spectrometer (HORIBA Jobin Yvon) equipped with an Ar⁺ laser. The Raman scanning range was set from 100 to 1000 cm^-1^. X-ray photoelectron spectroscopy (XPS) was employed to analyze the elemental composition and chemical states of the materials.

**2.3 Photothermal Performance Evaluation of Ru NCs**

The photothermal performance of water, L-Cys, and Ru NCs was evaluated under the same power density for 8 min of continuous irradiation. To assess the photothermal stability of Ru NCs, the samples were irradiated for 10 s in each cycle, followed by cooling to room temperature, and this process was repeated for three consecutive cycles. Infrared thermal imaging was employed to capture photothermal images and monitor real-time temperature changes.

The photothermal conversion efficiency (η) was calculated according to the following equation:

$$\text{η = }\frac{\text{hS(Tmax-Tsurr)-Q}\text{0}}{\text{I(1-10}\text{-A}\text{660}\text{)}}\text{ }$$

**2.4 Photoelectrocatalysis** **and Electrocatalysis of Ru NCs**

All photoelectrochemical measurements were performed on a CHI 650E electrochemical workstation (Shanghai Chenhua Instrument Co., Ltd.). A conventional three-electrode configuration was employed, consisting of a glassy carbon electrode (GCE, 3 mm in diameter) coated with the sample as the working electrode, a platinum wire as the counter electrode, and a saturated calomel electrode (SCE) as the reference electrode. The entire electrochemical cell, including the three electrodes, was vertically irradiated with a 660 nm light source. The electrolyte used was an aqueous 0.5 M Na_2_SO_4_ solution.

The preparation of the working electrode was as follows: the bare GCE was polished, then sequentially rinsed with deionized water and ultrasonically cleaned in nitric acid, acetone, ethanol, and deionized water, followed by drying at room temperature. Subsequently, Ru NCs were drop-cast onto the electrode surface and allowed to dry naturally. After fixation of the modified sample using a naphthol solution, photocurrent response and electrochemical impedance spectroscopy (EIS) measurements were conducted in Na₂SO₄ electrolyte.

Then, Ru NCs solutions with the same concentration were irradiated with a 660 nm light source for 1, 2, 3, 4, 5, 6, 7, and 8 min. The photocatalytic performance was evaluated using a UV–visible (UV–Vis) spectrophotometer.

**2.5 Bacteria culture**

**Bacterial Culture and Biofilm Formation**

*P. gingivalis* was cultured in tryptic soy broth (TSB), while *V. parvula* was grown in *Veillonella* culture medium. Single-species biofilms were formed in 24-well plates by inoculating with bacterial suspensions adjusted to 10^8^ CFU/mL. The samples were divided into four groups: Control, L-Cys, Ru NCs, and Ru NCs + L (with 660 nm laser irradiation) group. For dual-species biofilms, a co-culture system was established in 24-well plates using transwell inserts, with *P. gingivalis* (10^5^ CFU/mL) inoculated in the lower chamber and *V. parvula* (10^8^ CFU/mL)in the upper chamber. After 24 h of incubation at 37℃, the upper chamber received the following treatments: Control, Cys, Ru NCs, and Ru NCs + L ( with 660 nm laser irradiation）group. The biofilms were then cultured for an additional 3 days at 37℃.

**Standard Plate Counting Assay for Bacteria**

After 3 days of incubation, bacterial suspensions from the lower chambers of each group were collected, serially diluted, and analyzed using the standard plate counting method.

**Live/Dead Bacterial Staining Assay**

*P. gingivalis*, *V. parvula*, or the mixed suspension of *P. gingivalis* and *V. parvula* after different treatments were co-incubated with the corresponding samples in a 24-well plate containing glass slides for 72 h. Mature biofilms were then stained with fluorescent dyes in the dark at room temperature for 15 min, followed by three gentle washes with PBS. The biofilms were observed using a confocal laser scanning microscope (CLSM). Dead Bacteria exhibited red fluorescence, whereas live bacteria showed green fluorescence. Three-dimensional biofilm structures were captured and analyzed using ImageJ software.

**Fluorescence in Situ Hybridization (FISH) Imaging of Biofilms**

The biofilms were first washed with Cell and Protoplast Washing buffer (CPW) to remove non-adherent bacteria and fixed with 4% paraformaldehyde (PFA) in an ice bath for 3 h. After fixation, the samples were rinsed three times with cold PBS and air-dried at 46 °C for 15 min. The samples were then treated with lysozyme (1 mg mL^-1^) for 10 min to facilitate probe penetration, followed by dehydration in graded ethanol solutions of 50%, 80%, and 96% (v/v) for 3 min each. Finally, the samples were air-dried at 46 °C for 10 min.

Hybridization was performed in buffer containing 20% formamide. Fluorescent probes specific for *P. gingivalis* and *V. parvula* were added to the hybridization buffer (2 μL probe per 200 μL buffer), thoroughly mixed, and dropped onto the dehydrated samples. The hybridization was carried out at 46 °C for 1.5 h in the dark. After hybridization, the samples were washed in preheated washing buffer (48 °C) for 10–15 min, briefly rinsed with ice-cold deionized water for 3 s, and dried with compressed air.

The samples were then observed using a confocal laser scanning microscope (CLSM). For each group, five random fields of view were captured. Images (423.47 μm × 423.47 μm) were collected at 2 μm z-intervals under identical acquisition parameters. Image analysis was performed using ImageJ software(Fiji).

**Flow Cytometry Analysis**

To determine the relative proportions of *P. gingivalis* and *V. parvula* in the biofilms, flow cytometry analysis was performed. After treatment, the biofilms were washed with CPW buffer to remove planktonic bacteria. Subsequently, the biofilms were scraped from the culture wells and resuspended in PBS. To ensure the accuracy of flow cytometry detection, the samples were vigorously vortexed to dissociate the biofilm structure into single-cell suspensions. Bacterial cells were collected by centrifugation and fixed with 4% PFA for 3 h. After washing with PBS, the cells were treated with lysozyme for 10 min to enhance probe permeability, followed by dehydration in graded ethanol. For hybridization, the cell pellets were resuspended in hybridization buffer containing specific fluorescent probes and incubated at 46 °C for 1.5 h in the dark. After hybridization, the cells were collected by centrifugation, washed with preheated washing buffer to remove unbound probes, and finally resuspended in 500 μL of PBS. Detection was performed using a flow cytometer, and the proportions of each bacterial species were calculated using FlowJo software.

**Lactic acid**

Since *V. parvula* primarily utilizes lactate as a carbon source, residual lactate concentration in the culture medium was measured to evaluate the metabolic activity and viability of the bacteria. A higher residual lactate concentration indicates lower bacterial consumption, reflecting reduced bacterial viability. Following the treatments, the culture supernatants were collected from the upper chambers of the transwell system. The lactate concentration was quantified using a Lactic Acid Assay Kit (A019-2-1, Nanjing Jiancheng Bioengineering Institute, China) following the manufacturer's instructions. The absorbance was measured using a microplate reader. The lactate concentration (mmol/L) was calculated based on the standard curve equation:

*y*=10.58*x*−0.1016

‘y’ represents the lactate concentration and ‘x’ represents the absorbance value. Fresh culture medium was used as a negative control to determine the initial lactate baseline.

**PCR Detection of *P. gingivalis* Virulence Genes**

Primer design and synthesis：Specific primers targeting 16S rRNA, fimA, kgp, rgpA, and rgpB genes were designed based on published sequences and synthesized by Sangon Biotech Co., Ltd. (Shanghai, China). The primer sequences are listed below:

| Gene | Forward (5′→3′) | Reverse (5′→3′) |
| --- | --- | --- |
| 16S-rRNA | TGTAGATGACTGATGGTGAAA | ACTGTTAGCAACTACCGATGT |
| *fimA* | CAGCAGGAAGCCATCAAATC | CAGTCAGTTCAGTTGTCAAT |
| *Kgp* | AGCTGACAAAGGTGGAGACCAAAGG | TGTGGCATGAGTTTTTCGGAACCGT |
| *RgpA* | CTGCGAGCGGTATTAGTGGT | CTACCAGCCCGTTTCCAACT |
| *RgpB* | TCGGGACAAGTGTACGAACG | AACCAGTCTTGGGCTTCTCC |

Total bacterial RNA was extracted using Trizol reagent (Invitrogen, USA) according to the manufacturer’s instructions. Samples were collected, centrifuged at 6000 × g for 10 min, and lysed in Trizol. After phase separation with chloroform and RNA precipitation with isopropanol, the RNA pellet was washed with 75% ethanol, air-dried, and dissolved in DEPC-treated water. RNA purity and concentration were determined using a NanoDrop spectrophotometer (Thermo Fisher Scientific, USA), and all samples were adjusted to equal concentrations prior to reverse transcription. Complementary DNA (cDNA) was synthesized using the PrimeScript™ RT reagent Kit with gDNA Eraser (Takara, Japan) following the manufacturer’s protocol, and stored at −80 °C until use. Quantitative real-time PCR (qPCR) was performed with TB Green® Premix Ex Taq™ II (Takara, Japan) on a CFX96 Real-Time PCR Detection System (Bio-Rad, USA) using the following cycling conditions: 95 °C for 30 s, followed by 40 cycles of 95 °C for 5 s and 60 °C for 34 s, with a final melting curve analysis. Each reaction was performed in triplicate, and 16S rRNA served as the internal reference. Relative gene expression levels were calculated using the 2^−ΔΔCt method.

**Untargeted Metabolomics Analysis**

Non-targeted metabolomics was further performed following Ru NCs + L exposure. *V. parvula* was cultured anaerobically at 37 ℃ to mid‑log phase and treated with Ru NCs for 24 h, with irradiation (660 nm laser, 5 min/day) for three days. Cells were harvested by centrifugation (10,000 rpm, 4 ℃, 2 min), washed with cold PBS, and quenched with 80% methanol. Metabolites were extracted with methanol: acetonitrile: water (2: 2: 1, v/ v/ v), followed by ultrasonication and centrifugation. The supernatant was dried and reconstituted in 80% methanol for LC–MS/MS analysis.

Analysis was performed on a Thermo Fisher Vanquish UHPLC system coupled to a Q Exactive Orbitrap mass spectrometer. Data were acquired in positive and negative ionization modes. Raw data were processed using Compound Discoverer v3.3 for peak picking, alignment, and annotation against mzCloud and HMDB. Data were normalized to total ion current and log‑transformed. Multivariate analysis (PCA, OPLS‑DA) was conducted in SIMCA‑P v14.1. Differential metabolites were selected with VIP > 1.0 and *p* < 0.05 (Student’s *t*‑test). Pathway analysis was performed in R using clusterProfiler and KEGGREST, focusing on nitrogen metabolism, amino acid turnover, and redox pathways.

**Targeted Amino Acid Metabolomics Analysis**

*V. parvula* was incubated anaerobically at 37 ℃ to mid‑log phase under three conditions: Control, Ru NCs + L, and Ru NCs  + L + NO_3_^⁻^. The rescue group received 0.5 M sodium nitrate simultaneously. The samples were irradiated (660 nm, 5 min/day) for three days. Cells were harvested (10,000 rpm, 4 °C, 2 min), washed, and quenched with 80% methanol. Intracellular metabolites were extracted as above, and the supernatant was processed for LC–MS/MS.

Analysis used the same UHPLC‑Orbitrap platform. Amino acids were quantified by multiple reaction monitoring (MRM) with authentic standards. Data processing included peak detection and normalization in Compound Discoverer. Multivariate analysis used SIMCA‑P and MetaboAnalyst v5.0. Differential metabolites were identified with *p* < 0.05. KEGG pathway analysis was performed in R using KEGGREST, emphasizing nitrogen and amino acid metabolism, energy metabolism, and oxidative stress.

**Transcriptomic Analysis**

*P. gingivalis* ATCC 33277 was co‑incubated for 72 h at 37 °C, together with cell‑free supernatants from *V. parvula* pre‑treated under Control, Ru NCs + L, or Ru NCs  + L + NO_3_^⁻^ conditions. After biofilm formation, non‑adherent cells were removed. Biofilms were irradiated (660 nm, 5 min/day) for three days. Samples were collected, centrifuged (10,000 rpm, 2 min).

Total RNA was extracted with Trizol, and quality was assessed using NanoDrop and Bioanalyzer 2100. rRNA was removed (Zymo‑Seq RiboFree kit). cDNA libraries were constructed with random hexamers and the NEBNext Ultra RNA Library Prep Kit, and sequenced on a NovaSeq 6000 (PE150). Quality‑checked reads were aligned to the *P. gingivalis* genome using HISAT2, and counts were generated with featureCounts. Differential expression analysis was performed with DESeq2 (|log₂FC| > 1, adjusted *p* < 0.05). GO and KEGG enrichment analyses were conducted using clusterProfiler in R.

**2.6 Biosafety Evaluation**

**Biocompatibility Assessment of Ru NCs**

To evaluate the biocompatibility of Ru NCs, the cytotoxicity was assessed using a Cell Counting Kit-8 (CCK-8) assay with mouse fibroblast L929 cells. Specifically, L929 cells (100 μL) were seeded into 96-well plates at a density of 5 × 10^4^ cells/cm^2^ and cultured in Dulbecco’s Modified Eagle Medium (DMEM) supplemented with 10% fetal bovine serum (FBS) and 1% penicillin–streptomycin (100 U/mL penicillin and 100 μg/mL streptomycin). After 24 h of incubation, the medium was replaced with 100 μL of fresh medium containing 0, 100, 200, 300, 400, or 500 μg/mL of Ru NCs. The cells were then incubated at 37 °C under 5% CO_2_ for another 24 h. Subsequently, 10 μL of CCK-8 solution was added to each well containing 100 μL of culture medium, followed by incubation for 1 h. The absorbance was measured at 450 nm using a microplate reader.

**Cytoskeleton Staining and Cellular Morphology Observation**

To visualize cellular morphology and assess the impact of Ru NCs on cytoskeletal integrity, fibroblasts were seeded in confocal dishes and incubated with various concentrations of Ru NCs for 24 h. Following incubation, the cells were washed with PBS and fixed with 4% paraformaldehyde for 15 min. The cells were then permeabilized with 0.1% Triton X-100 for 5 min and blocked with 1% BSA to prevent non-specific binding.

The F-actin cytoskeleton was stained with Rhodamine Phalloidin for 30 min at room temperature in the dark. Subsequently, the cell nuclei were counterstained with DAPI (4',6-diamidino-2-phenylindole) for 5 min. After washing three times with PBS, the samples were imaged using a confocal laser scanning microscope to observe the cytoskeletal structure and nuclear morphology.

**Hematoxylin-eosin (H&E) staining and serum biochemical parameters**

To evaluate the potential systemic toxicity and histological changes associated with the bacterial infection and subsequent treatments, major organs (heart, liver, spleen, lungs, and kidneys) were harvested for histological analysis. Animals were maintained for 30 or 45 days post-treatment, after which they were euthanized for tissue. The harvested tissues were fixed in 4% paraformaldehyde, dehydrated, embedded in paraffin, and sectioned into 4-μm slices. The sections were stained with hematoxylin and eosin and examined under a light microscope to observe any pathological alterations.

For systemic biosafety evaluation, blood samples were collected from the Control and Ru NCs groups following 14 days of Ru NCs treatment. Key serum biochemical parameters, including liver and kidney function indicators, were measured using an automated biochemical analyzer to assess metabolic safety.

**2.7 Periodontitis Rat Model for *in vivo* Evaluation**

Details of the rat periodontal infection model establishment, treatment protocols, and histological analyses are provided in the main text.

**2.8 Statistical Analysis**

The statistical analysis methods are described in the main text.

**Supplementary Figures**


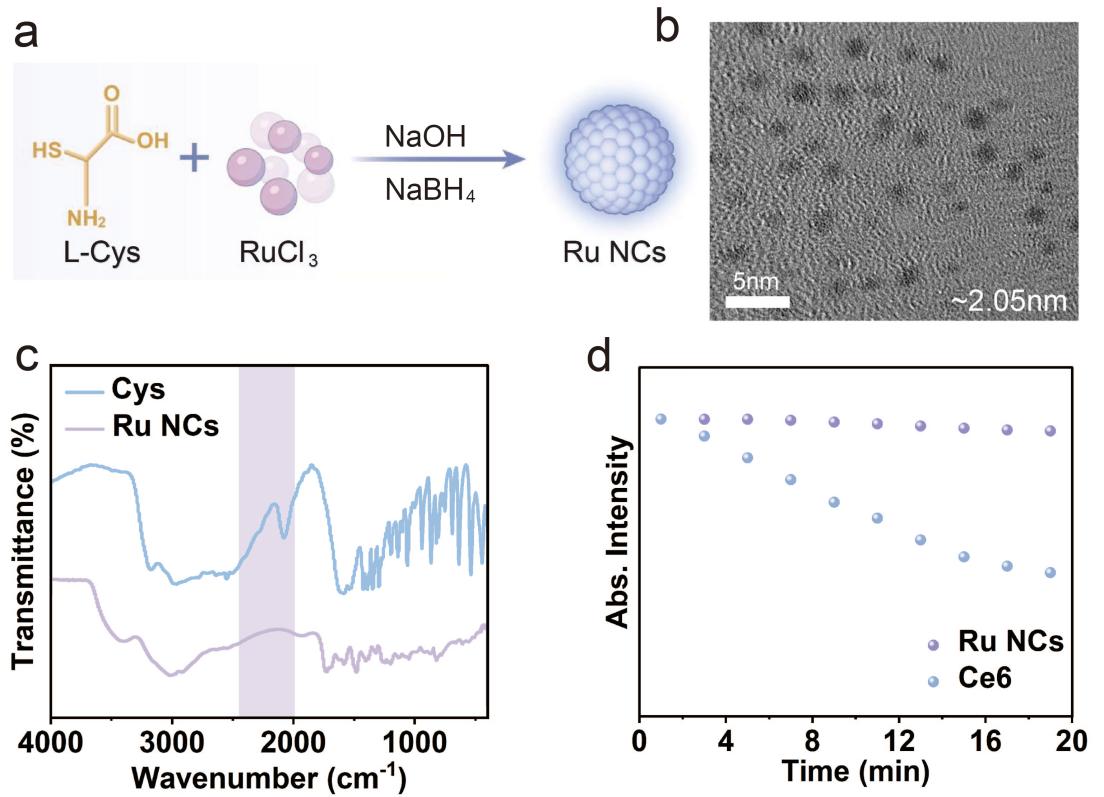


Figure S1. (a) Schematic diagram of the synthesis pathway for Ru NCs. (b) TEM images of Ru NCs. (c) FTIR spectra of Ru NCs. (d) The light stability of Ru NCs.


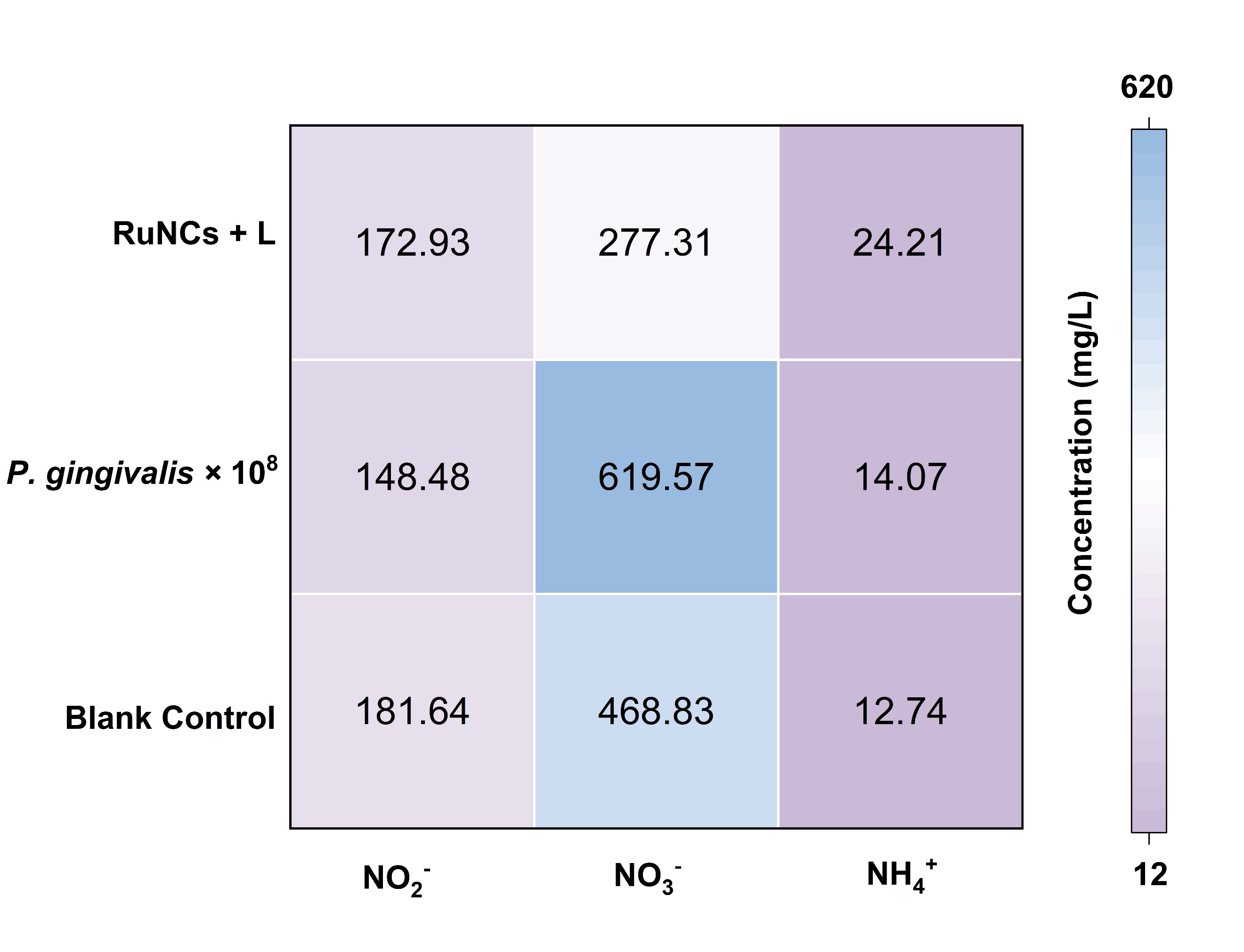


Figure S2. IC-MS quantification of NO_2_^⁻^, NO_3_^⁻^, and NH_4_^⁺^ in gingival crevicular fluid.


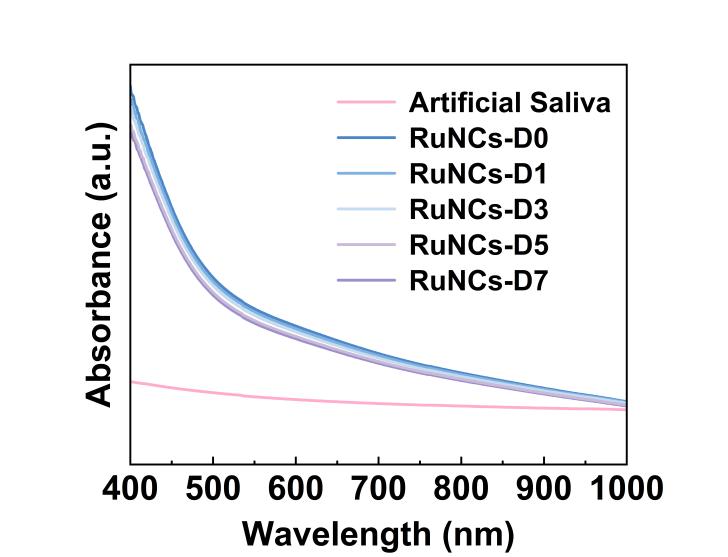


Figure S3. Long-term stability of Ru NCs in simulated oral environment.


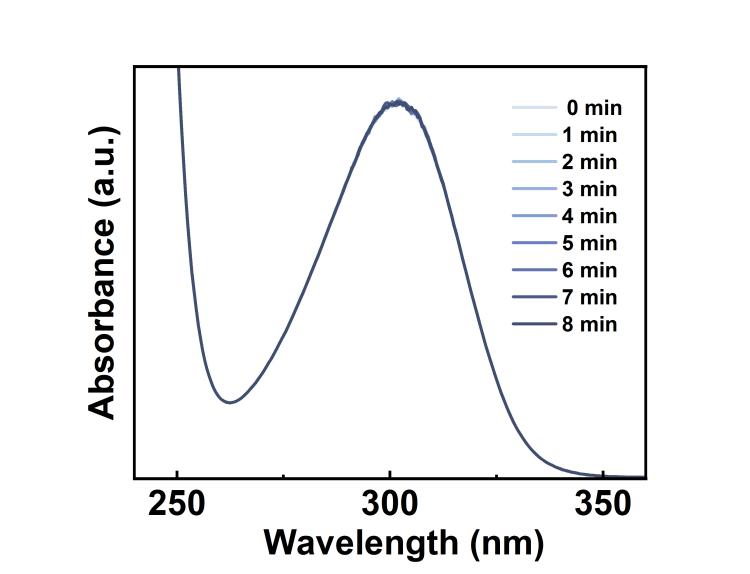


Figure S4. Time-dependent catalytic performance curve of Ru NCs (without laser).


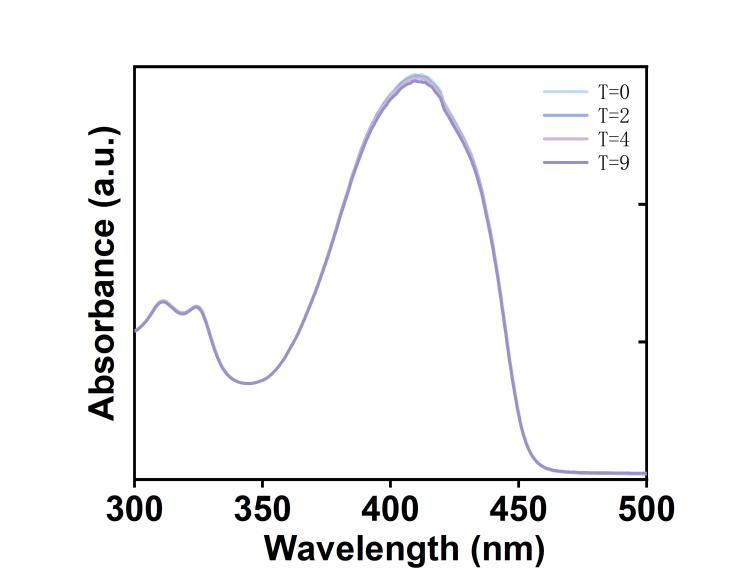


Figure S5. Detection of reactive oxygen species (ROS) generation by Ru NCs.


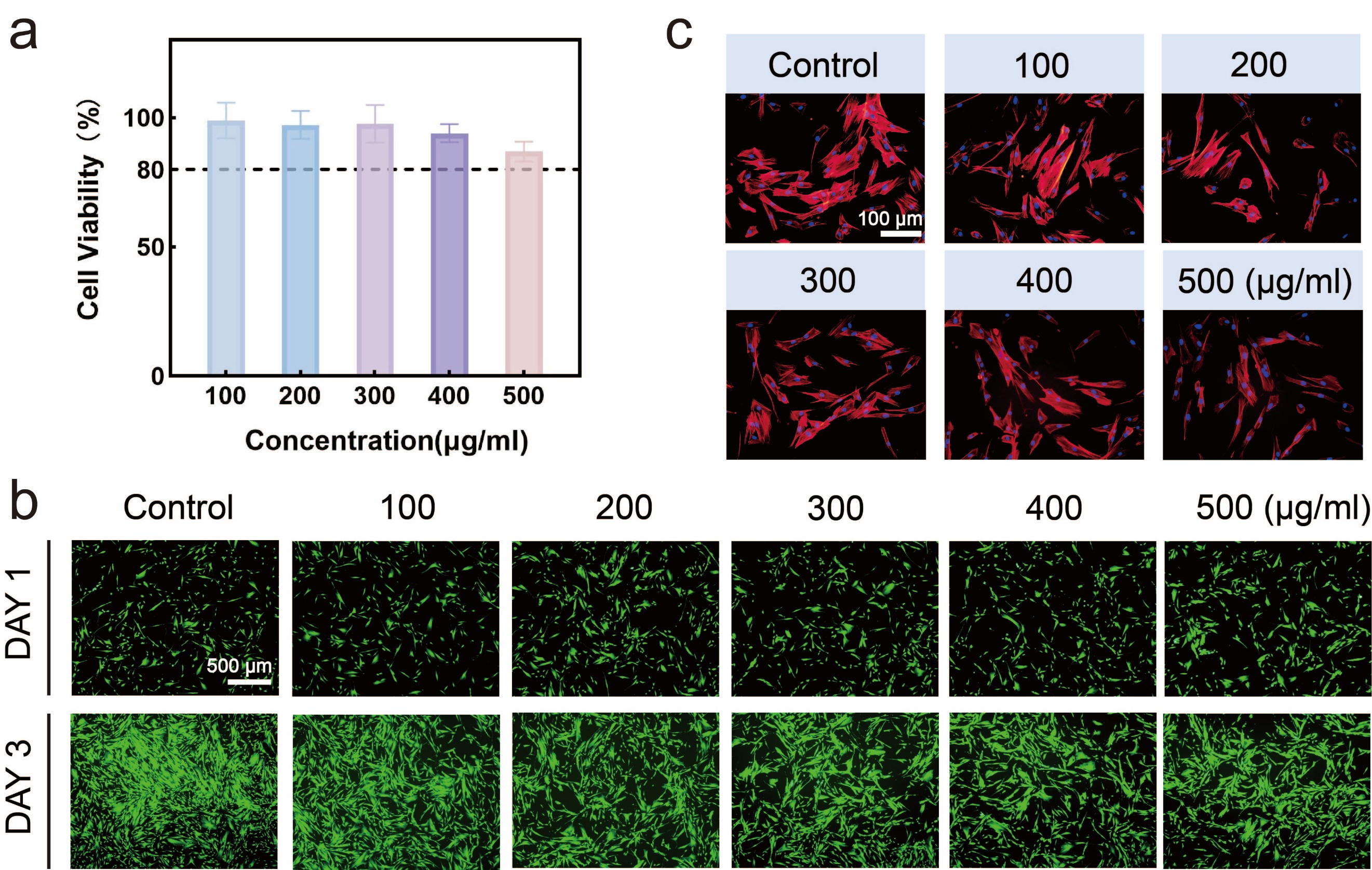


Figure S6. The biosafety of Ru NCs (a) CCK-8 assay results for Ru NCs at different concentrations. Data are presented as mean ± SD (n = 5) (b) Fluorescence images of L929 cells after treatment with different concentrations of Ru NCs. (c) Cellular skeleton for Ru NCs at different concentrations.


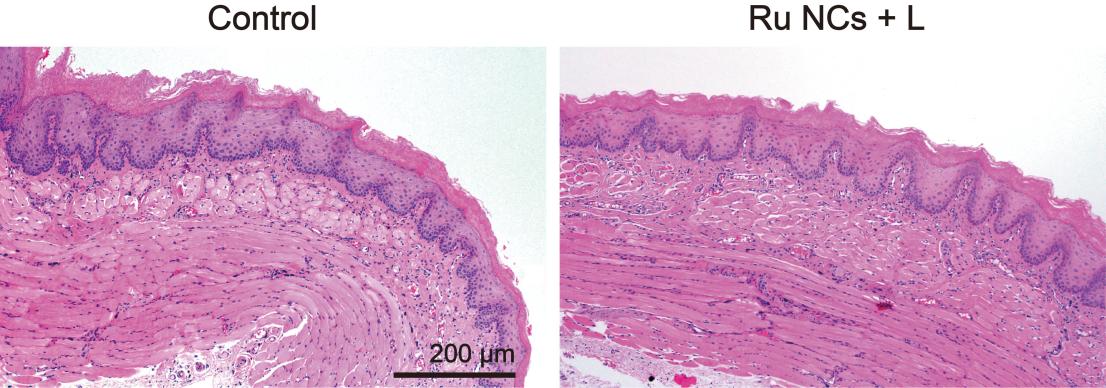


Figure S7. Histological analysis of buccal mucosa after photothermal treatment.


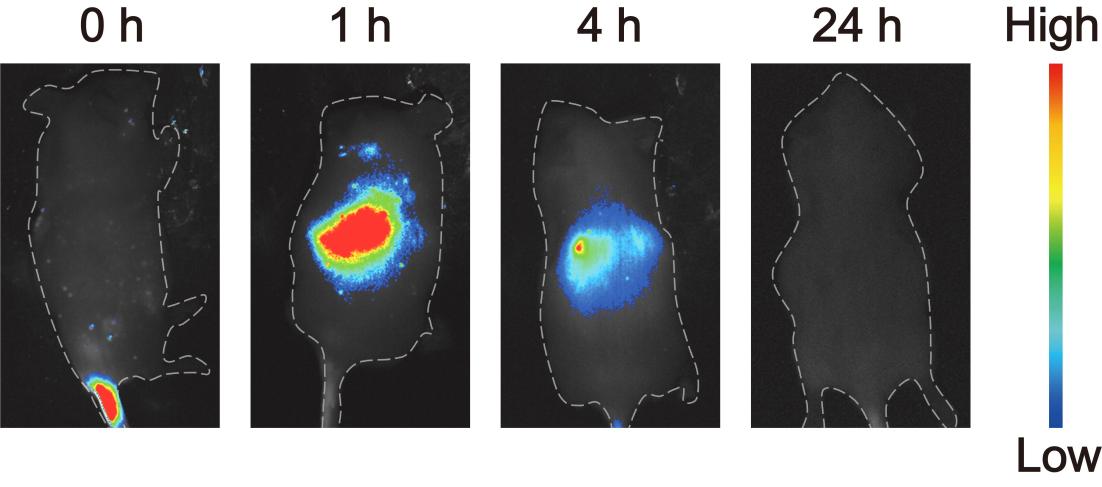


Figure S8. *In vivo* fluorescence imaging showing biodistribution and clearance of Ru NCs.


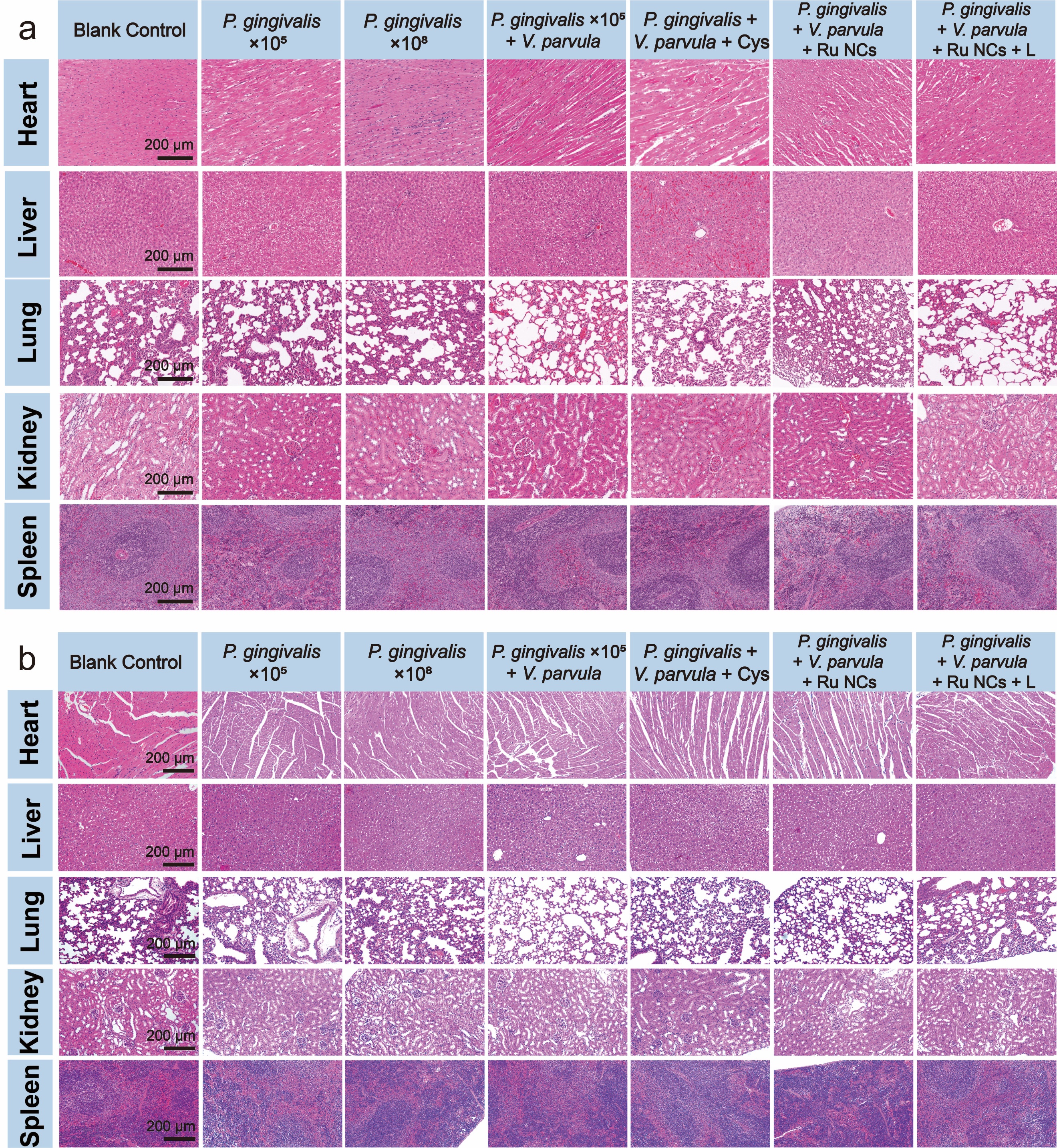


Figure S9. The biosafety of Ru NCs. (a) Typical H&E-stained images of major organs, including heart, liver, lung, kidney, and spleen, 30 days after Ru NCs treatment. (b) H&E staining images of major organs 45 days after Ru NCs treatment to assess systemic toxicity. Scale bar: 100 μm (n = 3 independent experiments).


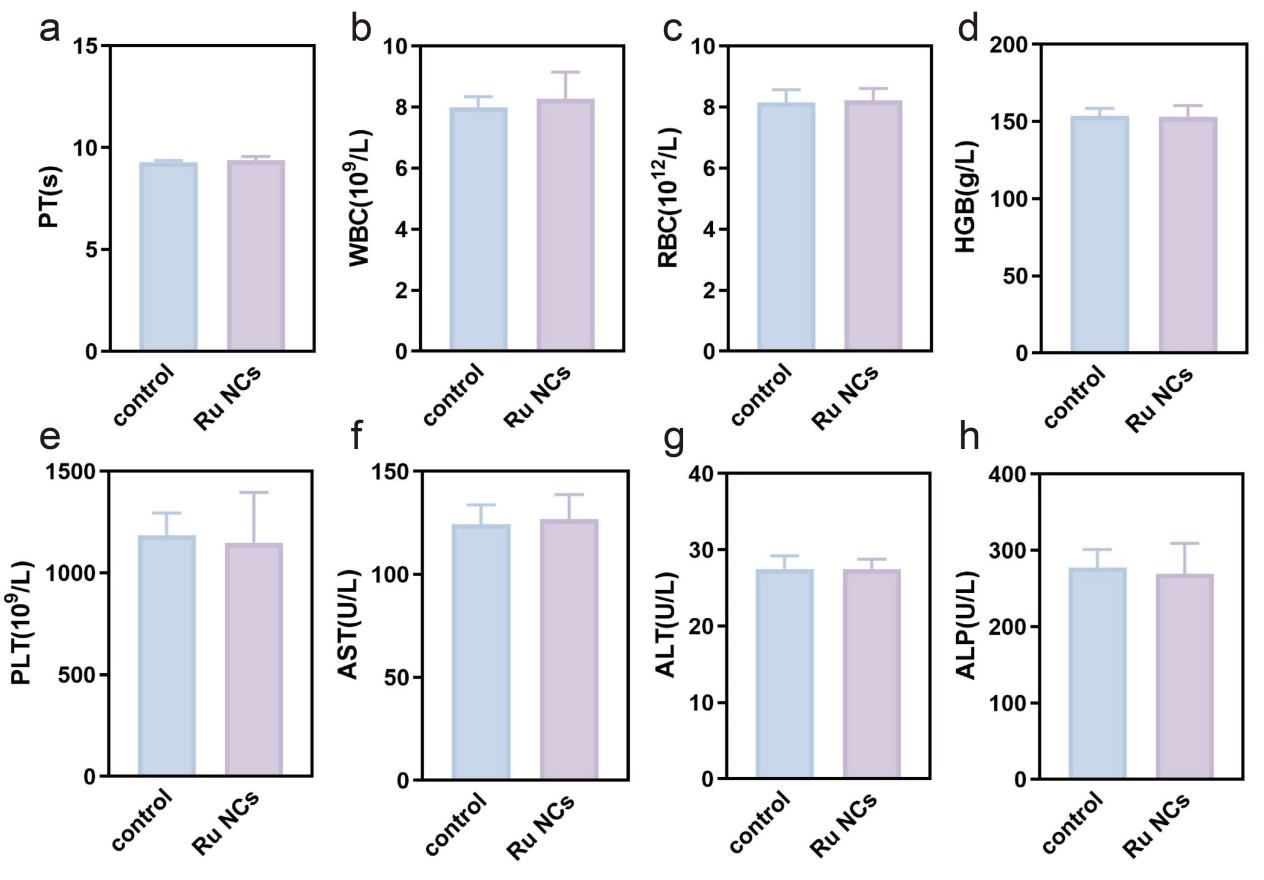


Figure S10. *In vivo* study of Ru NCs' effects on mouse liver and kidney function. Biochemical assays measured (a-b) coagulation, (c) white blood cells, (d) red blood cells, (e) hemoglobin, (f) platelets, and (g-i) liver function in mouse blood. Data are presented as mean ± SD (n = 4).


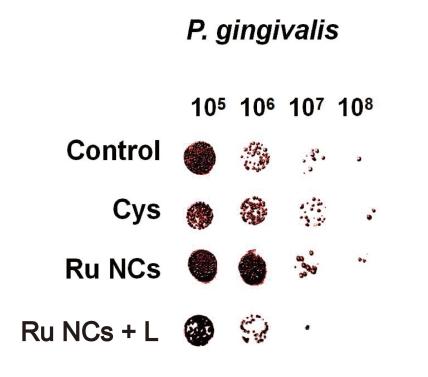

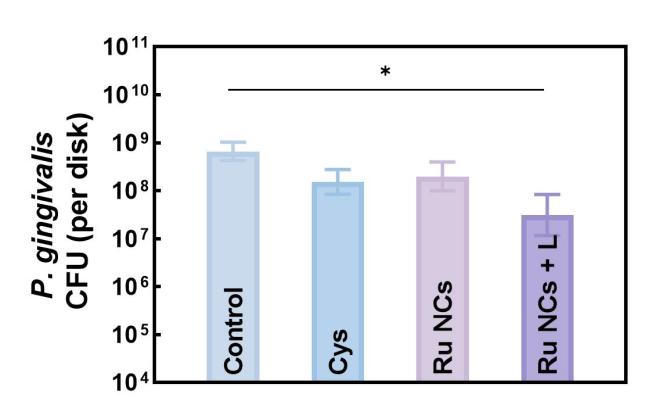


Figure S11. CFU Images and corresponding statistical information of *P. gingivalis* biofilms. (n = 3; **p* < 0.05, ***p* < 0.01, ****p* < 0.001; ns indicates no statistical significance).


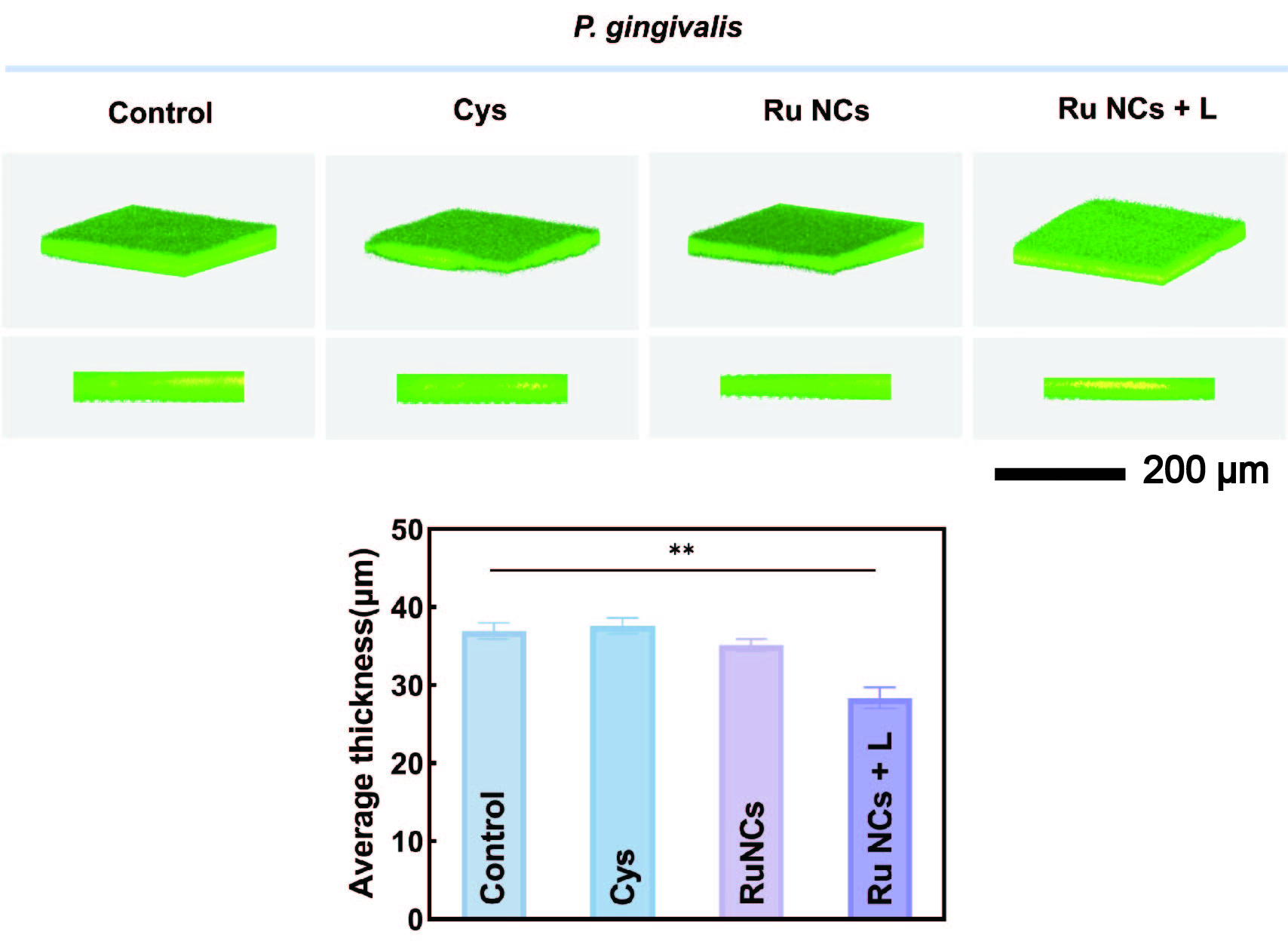


Figure S12. Representative three-dimensional live/dead staining images and fluorescence images of biofilm thickness in *P. gingivalis* biofilm. Data in panels are presented as mean ± SD (n = 3 biological replicates); statistical significance was evaluated using one-way ANOVA followed by Tukey's post-hoc test; **p* < 0.05, ***p* < 0.01, ****p* < 0.001; ns, not significant.


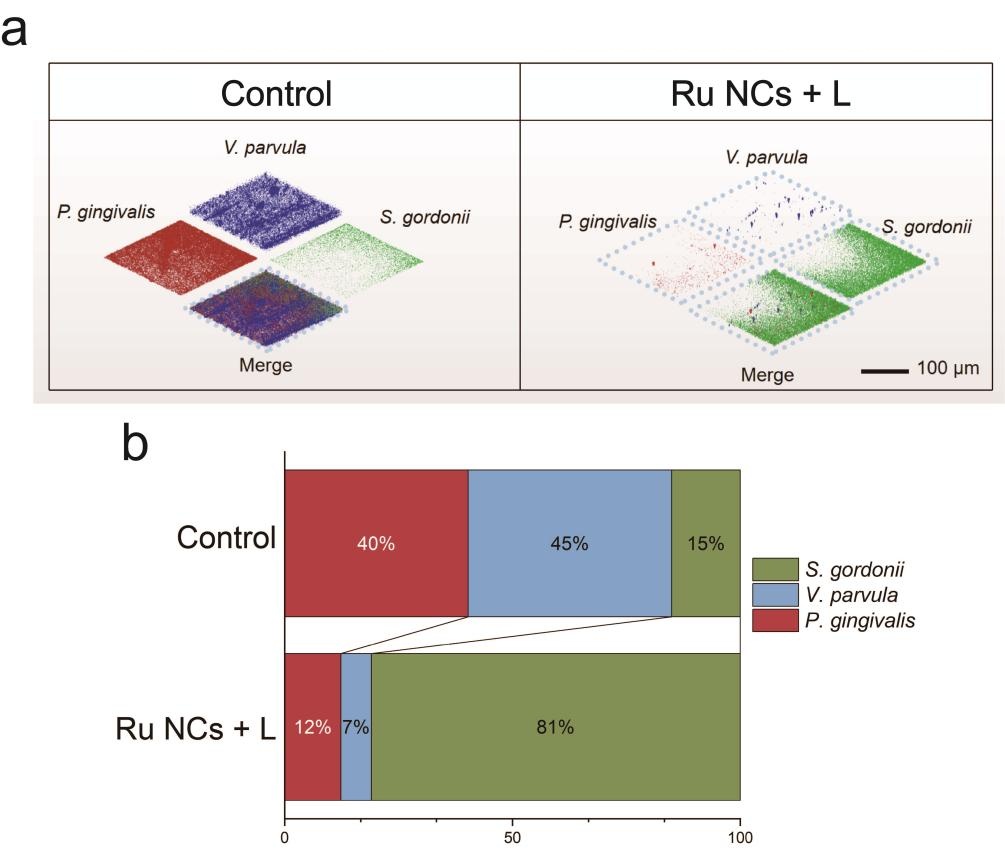


Figure S13. (a) FISH analysis showing selective inhibition of pathogenic bacteria by Ru NCs (*V. parvula*, red; *P. gingivalis*, blue; and commensal *S. gordonii*, green). (b) Statistical chart of the distribution of *V. parvula*, *P. gingivalis,* and *S. gordonii*.


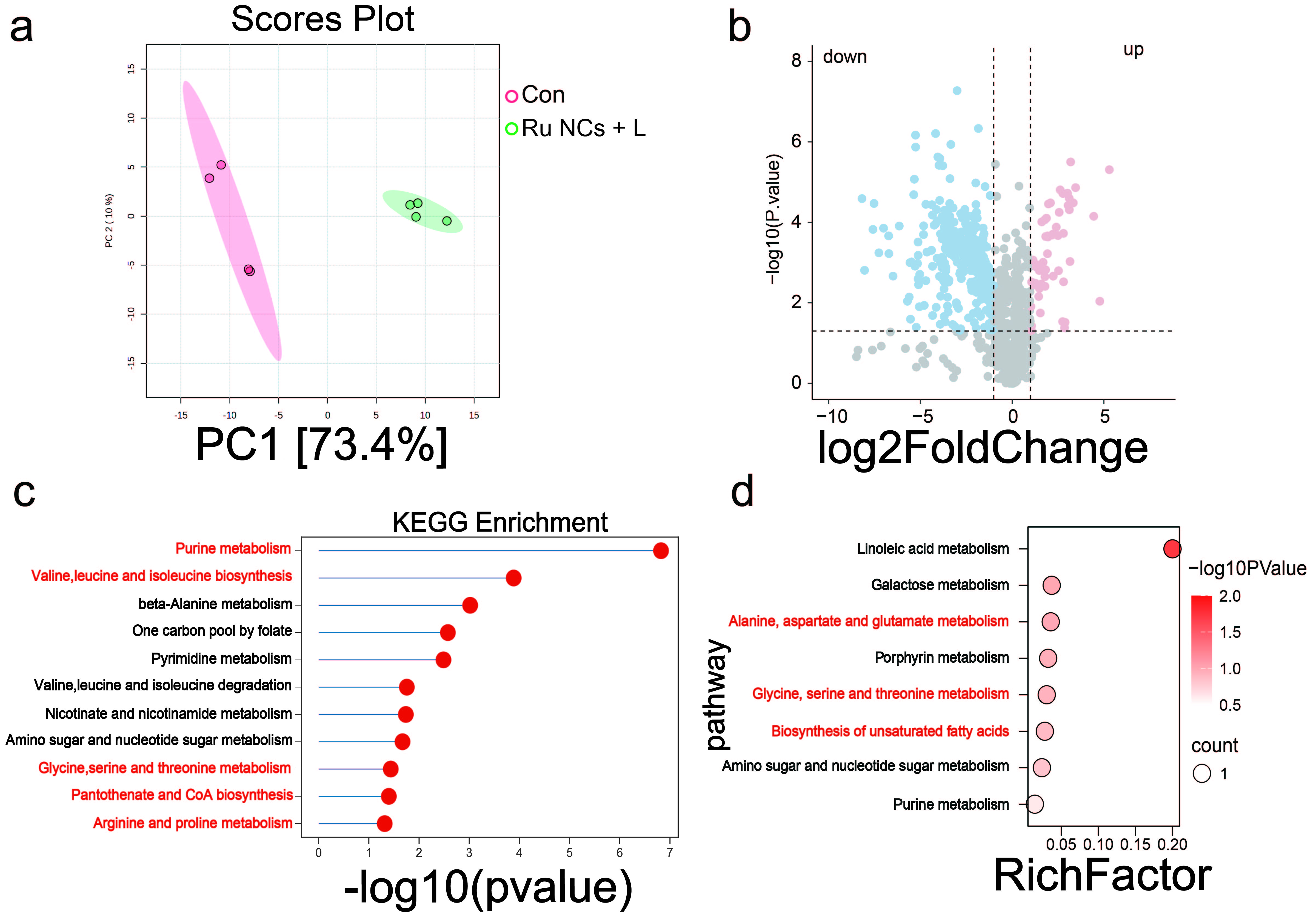


Figure S14. Multilevel characterization of non-targeted metabolomics of *V. parvula* under Ru NCs + L treatment. (a) Principal component analysis (PCA) score plot illustrating overall metabolic separation among groups. (b) Volcano plot showing the distribution of differential metabolites between the Control and Ru NCs + L-treated groups (p < 0.05, VIP > 1, |log2FC| > 1;n = 4 biological replicates). (c) KEGG pathway lollipop plot of enriched metabolic pathways identified by pathway analysis. (d) Over-representation analysis (ORA) of upregulated metabolites showing enriched metabolic pathways.





Figure S15. Targeted amino-acid metabolomics of *V. parvula* under Ru NCs + L and nitrate supplementation. (a–b) PCA score plots of metabolomic profiles for Control, Ru NCs + L, and Ru NCs + L + NO_3_^⁻^. (c) Volcano plot showing significantly altered metabolites between the Control and Ru NCs + L-treated groups (*p* < 0.05, |log_2_FC| > 1; n = 3 biological replicates). (d) Volcano plot showing significantly altered metabolites between the Control and Ru NCs + L + NO_3_^⁻^ groups under the same screening criteria. Lollipop plots of differential metabolites: (e) Control vs Ru NCs + L; (f) Control vs Ru NCs + L + NO_3_^⁻^. (g) Heatmap of differential metabolites in the Ru NCs + L group, highlighting alterations in amino-acid, nitrogen, and energy-metabolism-related pathways. (h) Pathway enrichment dot plot for the Ru NCs + L group based on differential metabolites. (i) Heatmap (Z-score) of differential metabolites for the Ru NCs + L + NO_3_^⁻^ group. (j–k) Integrated metabolite–pathway network maps built from differential metabolites and enriched pathways: (j) Ru NCs + L vs Control; (k) Ru NCs + L + NO_3_^⁻^ vs Control. (l-m) Quantification of L-Theanine and L-Tyrosine across the three groups (mean ± SD, n = 3; *p* < 0.05, one-way ANOVA with Tukey’s post-hoc test).


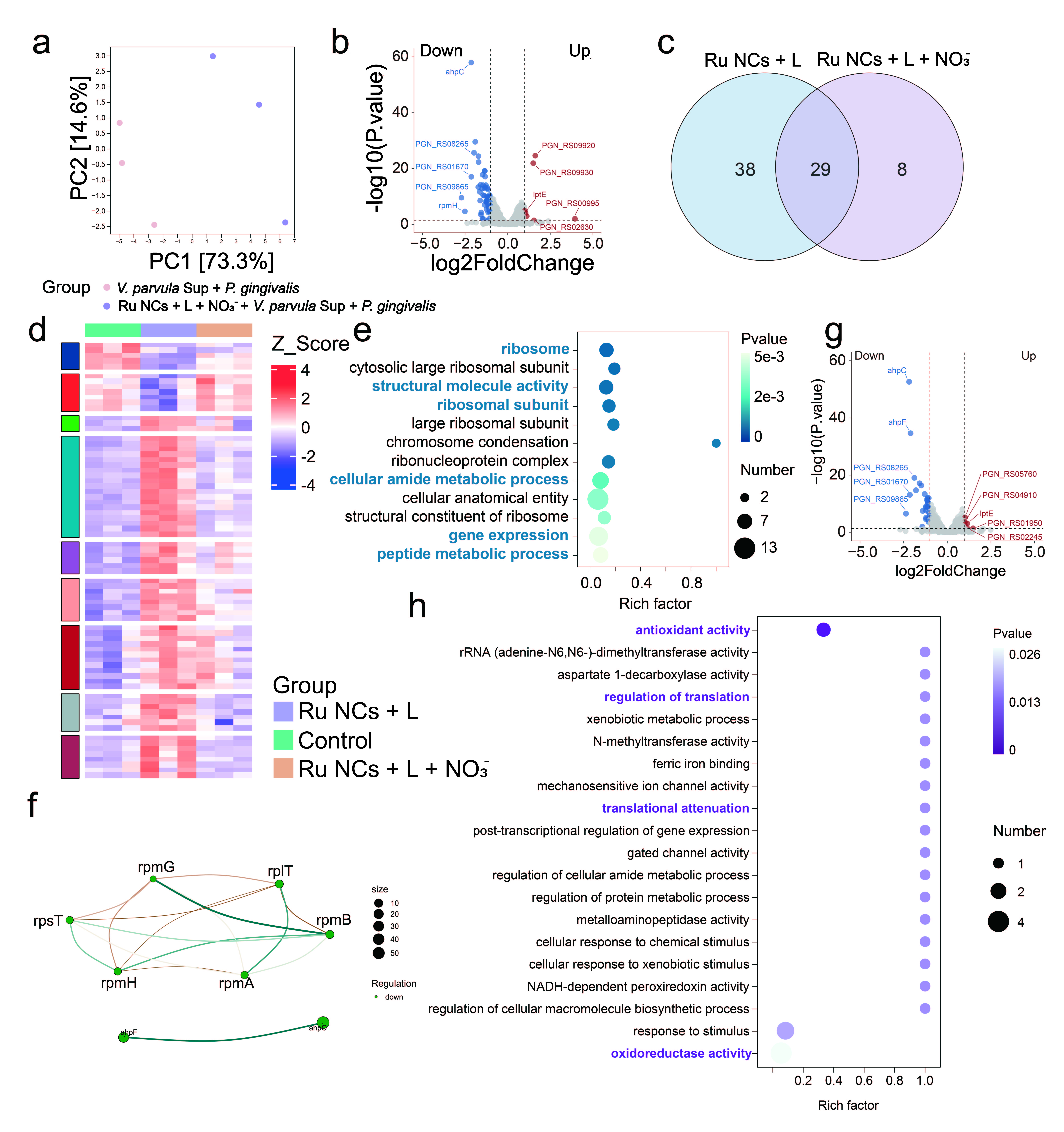


Figure S16. Transcriptomic analysis of *P. gingivalis* under Ru NCs + L treatment and nitrate supplementation. (a) PCA plots showing transcriptional profiles of the Control and Ru NCs + L + NO_3_^⁻^ groups. (b) Volcano plots showing differentially expressed genes (DEGs) between the Control and Ru NCs + L groups (p < 0.05, |log2FC| > 1; n = 3 biological replicates). (c) Heatmap (Z-score) of differentially expressed genes (DEGs) across the three groups. (d) Venn diagram showing the overlap of DEGs between the Ru NCs + L and Ru NCs + L + NO_3_^⁻^ groups. (e) Protein–protein interaction (PPI) network of representative ribosomal proteins in the Ru NCs + L group. (f) Volcano plots showing differentially expressed genes (DEGs) between the Control and Ru NCs + L + NO_3_^⁻^ groups (p < 0.05, |log2FC| > 1; n = 3 biological replicates). (g) Gene Ontology (GO) enrichment analysis of downregulated gene sets in the Ru NCs + L-treated group, highlighting envelope organization and translational repression. (h) GO enrichment analysis of downregulated genes in the Ru NCs + L + NO_3_^⁻^ group.
